# Bilayered skin equivalent mimicking psoriasis as predictive tool for preclinical treatment studies

**DOI:** 10.1038/s42003-024-07226-x

**Published:** 2024-11-18

**Authors:** Bianka Morgner, Oliver Werz, Cornelia Wiegand, Jörg Tittelbach

**Affiliations:** 1 University Hospital Jena, Department of Dermatology, Friedrich Schiller University Jena, Jena, Germany; 2 Department of Pharmaceutical/Medicinal Chemistry, Institute of Pharmacy, Friedrich Schiller University Jena, Jena, Germany

**Keywords:** Experimental models of disease, Molecular medicine

## Abstract

Psoriasis is a prevalent, inflammatory skin disease without cure. Further research is required to unravel dysregulated processes and develop new therapeutic interventions. The lack of suitable in vivo and in vitro preclinical models is an impediment in the psoriasis research. Recently, the development of 3D skin models has progressed including replicas with disease-like features. To investigate the use of in vitro models as preclinical test tools, the study focused on treatment responses of 3D skin replicas. Cytokine-priming of skin organoids induced psoriatic features like inflammation, antimicrobial peptides (AMP), hyperproliferation and impaired differentiation. Topical application of dexamethasone (DEX) or celastrol (CEL), a natural anti-inflammatory compound reduced the secretion of pro-inflammatory cytokines. DEX and CEL decreased the gene expression of inflammatory mediators. DEX barely affected the psoriatic AMP transcription but CEL downregulated psoriasis-driven AMP genes. Subcutaneous application of adalimumab (ADM) or bimekizumab (BMM) showed anti-psoriatic effects via protein induction of the differentiation marker keratin-10. Dual blockage of TNF-α and IL-17A repressed the inflammatory psoriasis phenotype. BMM inhibited the psoriatic expression of AMP genes and induced *KRT10* and cell-cell contact genes. The present in vitro model provides a 3D environment with in vivo-like cutaneous responses and represents a promising tool for preclinical investigations.

## Abstract (web summary)

Psoriatic human skin equivalents provide a 3D in vitro test environment with in vivo-like cutaneous responses to anti-inflammatory compounds or biological antibody therapy qualifying them as tools for preclinical treatment studies.

## Introduction

Among inflammatory diseases of the skin, psoriasis (PSO) represents a prevalent chronic condition. PSO affects 0.5–11% of the human population worldwide with a lower prevalence among children^1^. The aetiology still lacks a complete understanding of the primary cause but a composition of genetic and immunological factors has been identified. So far, PSO is classified as a T helper (Th) cell driven disease. The dermatitis is assumed to emerge from activated dendritic cells releasing IL-23, IL-12 and TNF-α, which lead to Th17, Th1 and Th22 polarization and expansion. These activated Th cells secrete IL-17 (Th17), IL-22 (Th17, Th22) or TNF-α/IFN-γ (Th1) and stimulate the inflammatory response of epidermal keratinocytes^2,3^. This triggers the keratinocyte production of antimicrobial peptides (AMP) like psoriasin (S100A7), beta-defensin-2 encoded by the *DEFB4A* gene, elafin (PI3), lipocalin-2 (LCN2) and cathelicidin (LL37/CAMP)^3,4^. Apart from antimicrobial effects, AMPs like S100A7 have a chemotactic activity towards neutrophils^5^. Permanent high amounts of AMPs can even lead to apoptosis induction and tissue damage^6^. In addition, the activated epidermal keratinocytes release pro-inflammatory cytokines and chemokines to attract immune cells like neutrophils and macrophages^7,8^. A feedback loop is mediated via keratinocyte-released cytokines like TNF-α, IL-1β, IL-6 und IL-8 stimulating dendritic cells and recruiting Th17 cells, thus, sustaining the elevated Th17 milieu^9,10^. Furthermore, downstream molecules of the NFκB pathway like COX2 are induced in psoriatic lesions^11^. Apart from the production of AMPs and inflammatory mediators, Th-derived cytokines like IL-22, IL-17 and TNF drive keratinocyte proliferation^3^. Furthermore, differentiation processes and the production of structural proteins like filaggrin (FLG) or keratin-10 (CK10 encoded by *KRT10* gene) are impaired due to the psoriatic cytokine signalling, especially upon IL-22^12^. Decreasing CK10 levels are provisionally compensated by an upregulation of CK16, a keratin that is associated with inflammation, hyperproliferation and barrier impairments^13^. Thus, it is typically found in psoriatic skin^14^. Hyperproliferation and differentiation defects result in histological manifestations like epidermal thickening (acanthosis), a thicker *stratum corneum* (hyperkeratosis) with nuclear retention (parakeratosis) and elongated rete ridges. Immune dysregulation leads to neutrophil infiltrates accumulating in the *stratum corneum* (Munro’s microabscess)^15^.

Despite the knowledge of involved pathways in PSO onset and progression, the disorder cannot be cured permanently. Due to the multifactorial character of the dermatosis affecting inflammation, proliferation and differentiation processes in the skin, the treatment may fail due to disease heterogeneities in patients^16^. Hence, a lifetime disease management and therapeutic adaptions are necessary to maintain a certain life of quality. Mild-to-moderate forms of PSO are usually treated with topical application of corticosteroids. Glucocorticoid therapies are often combined with dithranol or vitamin D analogues like calcipotriol. In moderate-to-severe cases, systemic treatments are prescribed using methotrexate, cyclosporine A, fumaric acid or retinoids^17,18^. Modern treatment options include the usage of therapeutic antibodies when traditional systemic therapies fail or are contraindicatory. In PSO treatment, TNF-α inhibitors like infliximab or adalimumab, IL-23A antagonists like risankizumab or IL-17 blockers e.g. secukinumab have been approved^19^. With bimekizumab, a new biological for dual neutralization of IL-17A and IL-17F has recently been introduced to the market of therapeutic antibodies for PSO treatment^20^. Still, the development of new drugs and therapeutic compounds is of interest to cope with inflammatory skin diseases. Especially non-corticosteroid alternatives for topical applications are limited. Retrospective researches focus on natural compounds that have been known for their efficacy and usage in historic treatments. An example is the traditional Chinese medicine, which employs root extracts of the *Tripterygium wilfordii* Hook F plant to treat inflammatory conditions including PSO^21^. Celastrol (CEL) is one of the bioactive molecules that can be extracted from the medicinal plant. CEL is of therapeutic interest due to its anti-inflammatory and anti-proliferative properties^22–26^. In addition, the compound has already been shown to alleviate psoriatic conditions^27,28^.

Modern tissue research includes the in vitro generation of reliable in vivo-like skin substitutes. Various skin models have been established mimicking features of inflammatory diseases like psoriasis (PSO)^29–34^. These vary in their anatomical composition, cellular and matrix origin and/or way of disease induction. However, the suitability as preclinical models and the testing of medical compounds in these artificial tissues is still in the beginning. On the other hand, psoriasiform mouse models generated by chemical irritation or genetic manipulation are still widely used instead of in vitro models^35^. In addition to ethical issues, one has to be aware of anatomical, transcriptomic and especially immunological differences of murine skin compared to human skin^36,37^. The development of inflammatory skin diseases like PSO is limited to the human skin and does not emerge naturally in mice^38^. Considering this fact, rodents do not necessarily comply with the requirements of a suitable animal model for inflammatory skin disorders. Hence, it is of utmost importance to verify the suitability of in vitro models as preclinical test tools for new drug medications and therapeutic approaches as well as reveal their limitations. Knowing the opportunities and limitations of these models is essential for medical scientists to choose the appropriate in vitro substitute that complies with the research aim.

**Fig. 1:**
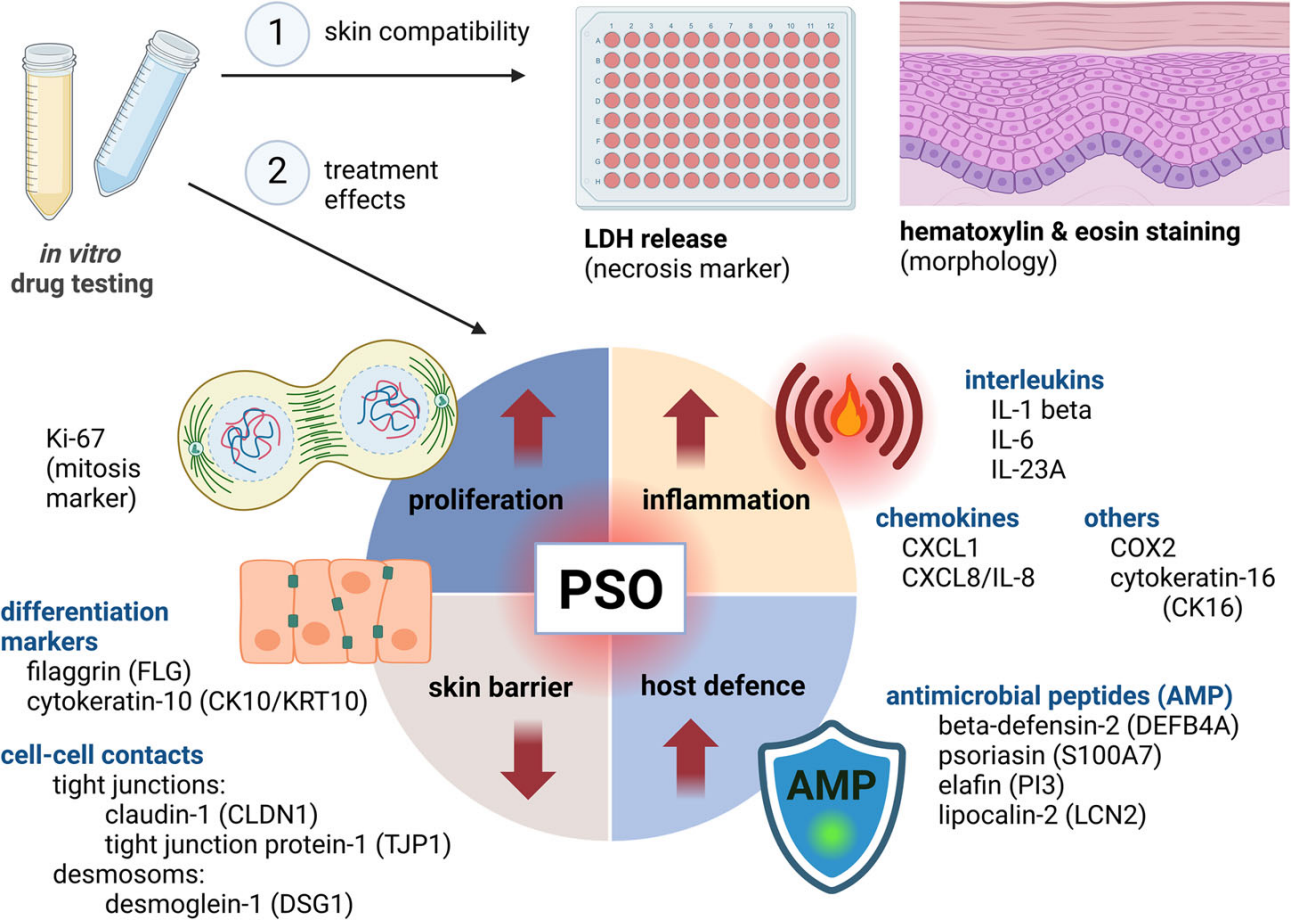
Markers for in vitro drug testing investigated in this study. Test substances were screened for their skin compatibility in 3D skin models via monitoring of lactate dehydrogenase (LDH) release and morphological evaluation by screening HE stained sections of the skin models. In the second step, anti-psoriatic activities of the compounds were investigated. PSO is characterized by hyperproliferation, inflammation, increased AMP expression and a weakened cutaneous barrier. Depicted markers were chosen to assess the efficacy of the test compounds to alleviate these hallmarks of PSO. Created in BioRender. Reddersen, K. (2024) https://BioRender.com/y54q683.

In this study, a 3D in vitro model mimicking psoriatic skin was used, which has been introduced recently ^32^. Here, the pathological signalling of Th cells and subsequent cascades was simulated by subcutaneous cytokine administration using recombinant IL-17A, IL-22, IL-6, IL-1α, TNF-α for PSO induction. As shown by our previous study, sole inflammatory Th1 (TNF-α + IFN-γ) priming does not induce typical PSO features like barrier weakening or differentiation defects with parakeratosis formation. This underlines the necessity of Th17 cytokines like IL-17A and IL-22 for PSO induction^32^. IL-6 is elevated in psoriatic skin and serum. Thus, the cytokine not only indicates inflammatory activity but also contributes to epidermal hyperproliferation^39,40^. IL-1α mainly accounts for loss of the differentiation marker keratin-10 as shown by Rabeony et al.^12^. PSO is a multifactorial disorder involving the dysregulation of various cytokines^41^. Hence, a multifactorial cytokine priming was chosen to induce the disease. The model includes a dermal compartment with fibroblasts and a differentiated epidermis generated from keratinocytes. This bilayered construction not only mimics the cutaneous anatomy comprising dermis and epidermis but also allows para- and intercellular communication. A crosstalk between fibroblasts and keratinocytes is essential for skin homeostasis and epidermal differentiation^42^. Despite the epidermis being the mainly affected layer upon PSO, dermal fibroblasts have been shown to be involved in the disease, too^43^. Furthermore, in this self-assembly model, the collagen deposition in the dermis is solely derived from the present fibroblasts without addition of animal collagen. The psoriatic human skin equivalent (PSO-HSE) has been shown to feature characteristic disease attributes of the inflammatory skin disorder^32^. The present study focussed on the responsiveness of such skin equivalents within a short period and aimed to illuminate the suitability of this skin model as preclinical test tool using topical and biological approaches. The study investigated the skin compatibility and effects of the test substances on inflammation, host defence via AMP modulation, differentiation and cellular adhesion. Figure 1 provides a systematic overview of all tested markers presented in this study and their relevance in PSO.

## Results

### Inherent recovery potential and responsiveness range of PSO-HSE

**Fig. 2:**
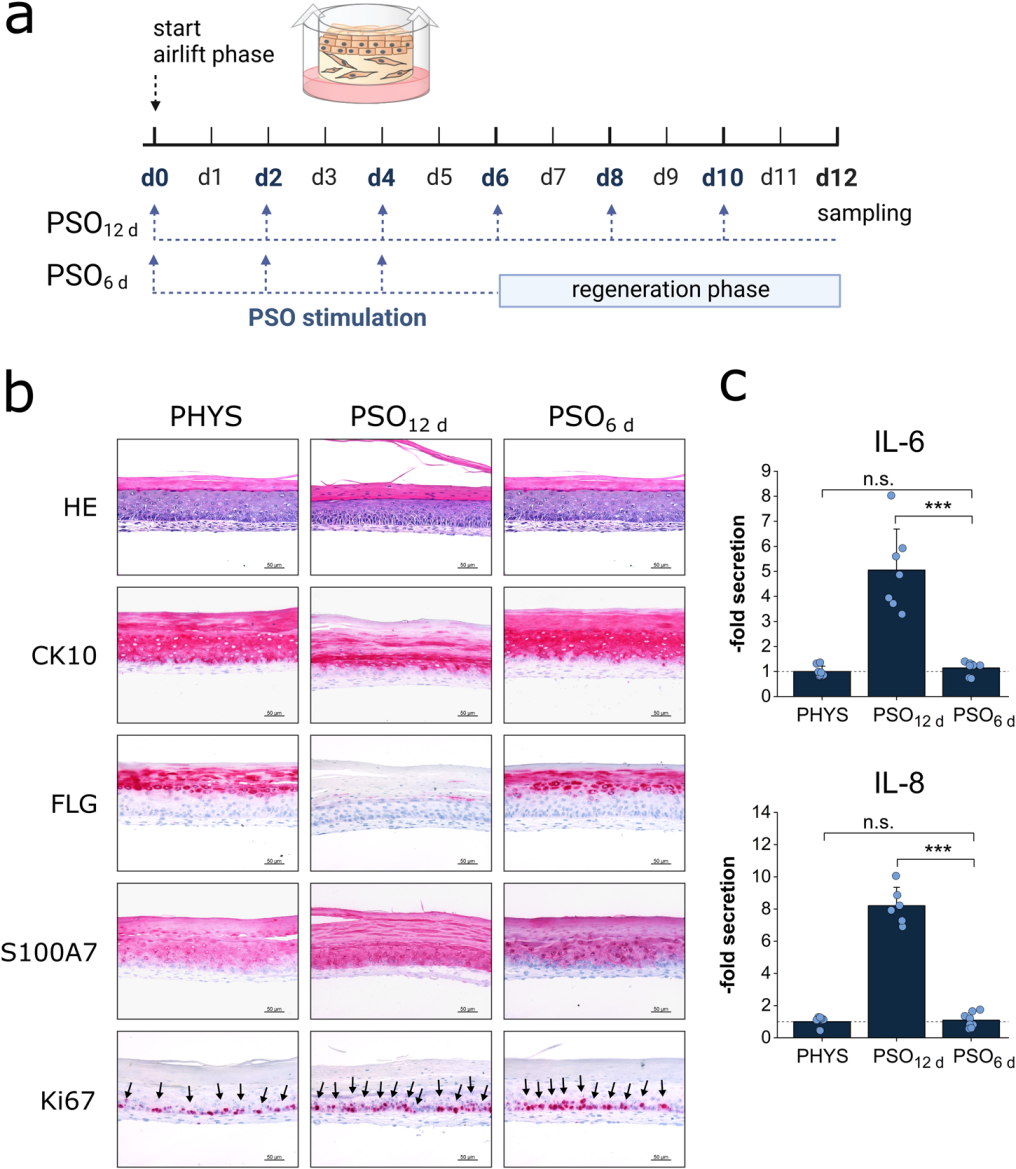
Regeneration ability of PSO-HSE on protein expression and cytokine secretion. **a** 3D skin equivalents were cultivated physiologically (PHYS) or cytokine-primed for psoriasis (PSO) induction during airlift incubation for 12 days (PSO_12 d_) or for 6 days followed by 6 days without cytokine stimulation (PSO_6 d_) to evaluate the regenerative potential. Blue arrows indicate the time points of the medium exchange and priming with the cytokine cocktail while the dotted lines represent the presence of the cytokines in the medium reservoir. Created in BioRender. Reddersen, K. (2023) https://BioRender.com/q43s554**b** Morphology and protein production of the differentiation markers keratin-10 (CK10), filaggrin (FLG), the antimicrobial peptide psoriasin (S100A7) and the mitosis marker Ki67 were analysed by HE and IHC. The protein of interest is stained in red by IHC processing. Ki67 positive basal cells were highlighted by black arrows. Scale bar: 50 µm, representative pictures of *n* = 2 skin equivalents. (**c**) Cytokine secretions of pro-inflammatory IL-6 and IL-8 in the undernatants of the organoids at day 12 were measured by ELISA. Statistics: Mann–Whitney *U* test; *p* ≤ 0.05 *, *p* ≤ 0.01 **, *p* ≤ 0.001 ***,*n* = 4 skin equivalents measured in technical duplicates.

3D skin equivalents displaying disease features of psoriasis (PSO) were generated upon cytokine stimulation mimicking the pathological T cell signalling causative for the disease onset. Being able to revert to a physiological phenotype is a prerequisite of in vitro models to serve as predictive tools for treatment studies. In order to investigate the potential of the in vitro generated PSO-HSE to revert to a physiological state, a regeneration test was performed. The models were cytokine PSO-primed for 6 days followed by 6 days without the pathological cytokine stimulation for regeneration (Fig. 2a). The HE staining revealed a microanatomy of the regenerated PSO_6 d_-HSE similar to physiological samples. While the PSO_12 d_-HSE featured a severe form of parakeratosis, the regenerated skin models seemed to have a lower number of nuclei present in the *stratum corneum*. Structural proteins like CK10 and FLG, which were strongly decreased after 12 days of PSO stimulation, were completely restored when the stimulus was removed at day 6. In contrast, the high levels of S100A7 and Ki67 remained after a regeneration phase of 6 days (Fig. 2b). Fully stimulated PSO_12 d_-HSE secreted elevated levels of the pro-inflammatory cytokines IL-6 and IL-8. The inflammatory feature via secretion of IL-6 and IL-8 by the PSO_6 d_ models, however, completely normalized to a physiological level (Fig. 2c).

**Fig. 3:**
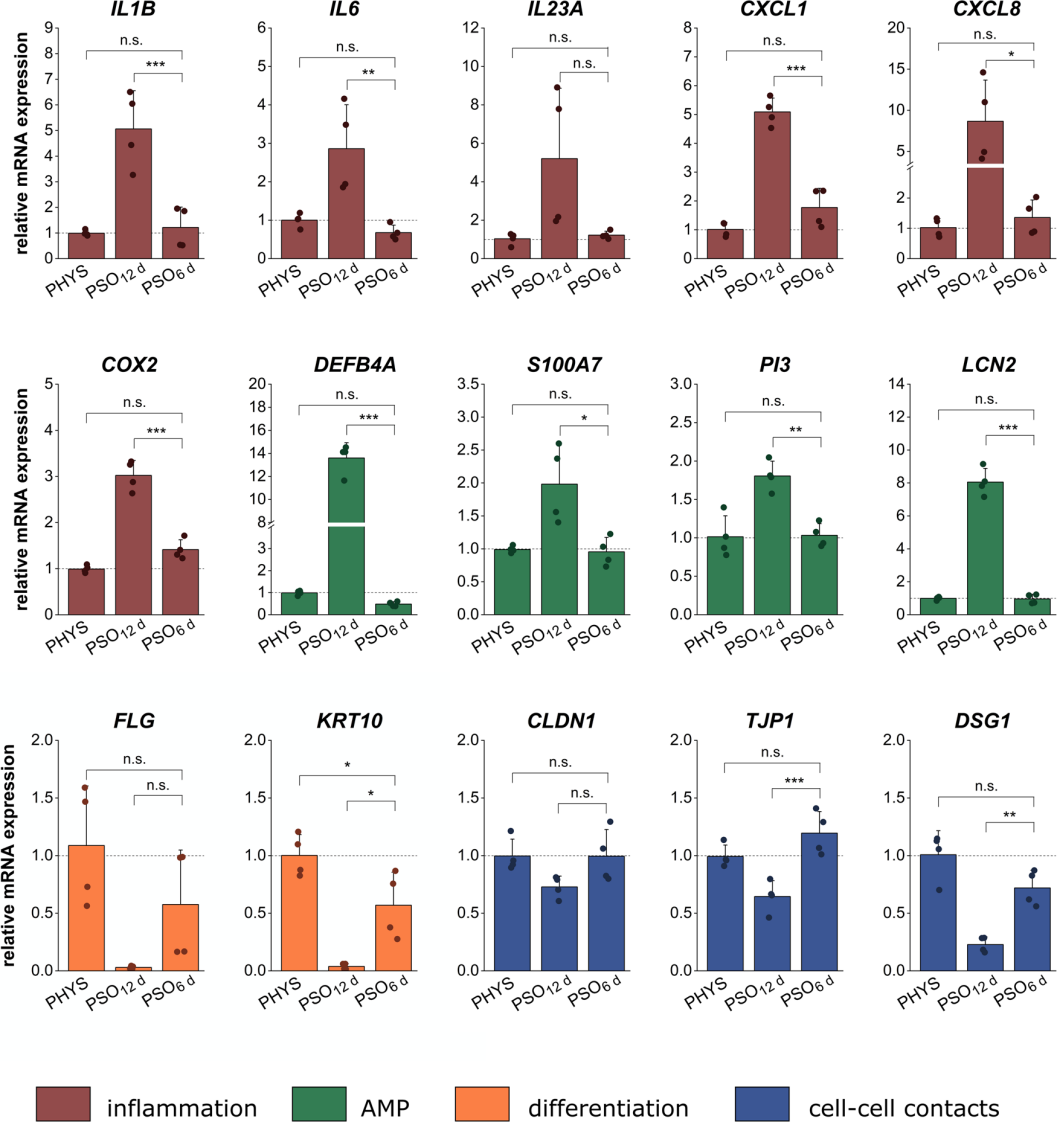
Regeneration ability of PSO-HSE on transcriptional levels. 3D skin equivalents were cultivated physiologically (PHYS) or cytokine-primed for psoriasis (PSO) induction during airlift incubation for 12 days (PSO_12 d_) or for 6 days followed by 6 days without cytokine stimulation (PSO_6 d_) to evaluate the endogenous regenerative potential. Gene expression was analysed via qPCR. The mRNA levels of genes involved in inflammation (red) like interleukin 1β (*IL1B*), interleukin 6 (*IL6*), interleukin 23 A (*IL23A*), chemokine (CXC motif) ligand 1 (*CXCL1*), chemokine (CXC motif) ligand 8 (*CXCL8*) and cyclooxygenase-2 (*COX2*), antimicrobial peptide (AMP) production (green) like beta-defensin-2 (*DEFB4A*), psoriasin (*S100A7*), elafin (*PI3*) and lipocalin-2 (*LCN2*), differentiation (yellow) like filaggrin (*FLG*) or keratin-10 (*KRT10*) and cell-cell contact formation (blue) via claudin-1 (*CLDN1*), tight junction protein-1 (*TJP1*) and desmoglein-1 (*DSG1*) were analysed. Statistics: one-way ANOVA with Bonferroni post hoc test; *p* ≤ 0.05 *, *p* ≤ 0.01 **, *p* ≤ 0.001 ***, *n* = 2 skin equivalents measured in technical duplicates.

In addition, regenerative effects were detected on transcriptional levels. PSO_12 d_-HSE showed characteristic pathological gene expression patterns, which were reverted towards physiological transcript levels after a 6 day regeneration phase (Fig. 3). While fully stimulated PSO_12 d_-HSE displayed high mRNA levels of *IL1B*, *IL6*, *IL23A*, *CXCL1*, *CXCL8* and *COX2*, these inflammatory genes were completely normalized to physiological expression levels in the PSO_6 d_-HSE. For these, no significant deviations to the PHYS controls were obtained. The AMP gene expression of *DEFB4A*, *S100A7*, *PI3* and *LCN2* was potently increased in PSO_12 d_-HSE but downregulated to a healthy baseline level in the regenerated equivalents. Genes encoding structural proteins/differentiation markers like *FLG* and *KRT10* were typically downregulated in the PSO_12 d_-HSE compared to physiological skin equivalents. After 6 days of regeneration, these genes were upregulated, even though the levels still tended to be marginally lower than physiological expression values. The PSO_12 d_-HSE showed a loss in the expression of the cell-cell contact genes *CLDN1*, *TJP1* and *DSG1*. Without the PSO stimulus for 6 days, the cellular adhesions genes showed a recovery of their expression levels comparable to physiological conditions (Fig. 3).

### Skin compatibility of topical treatment approaches

**Fig. 4:**
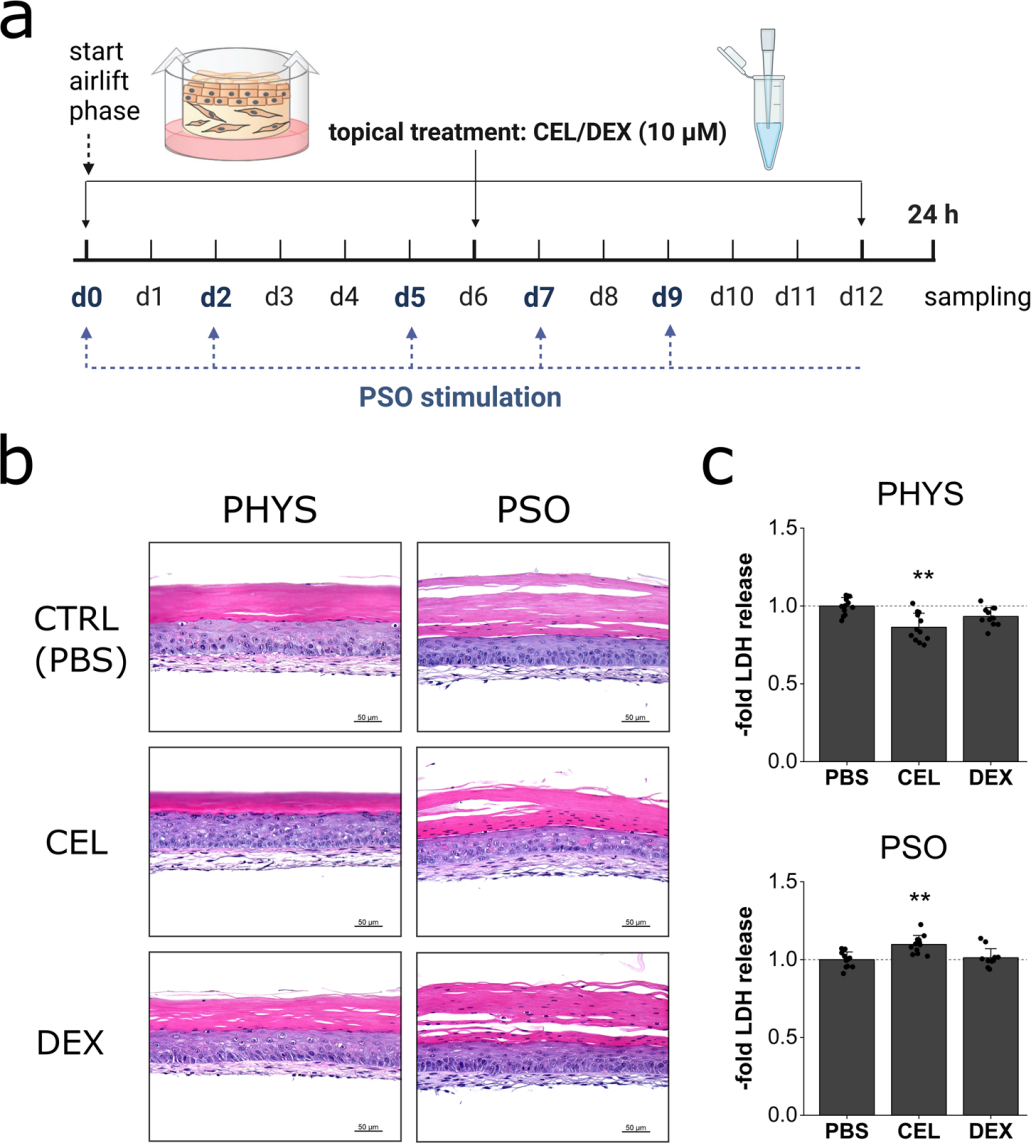
In vitro skin compatibility of topical drugs for treatment of PSO-HSE. **a** 3D skin equivalents were cultivated physiologically (PHYS) or cytokine-primed for psoriasis (PSO) induction and celastrol (CEL) or dexamethasone (DEX) were applied topically at day 0, 6 and 12 of airlift incubation. Blue arrows indicate the time points of the medium exchange and priming with the cytokine cocktail while the dotted line represents the presence of the cytokines in the medium reservoir. Created in BioRender. Reddersen, K. (2023) https://BioRender.com/s48k187. **b** HE staining was used to reveal putative cytotoxic effects via morphological changes after treatment of PHYS or PSO equivalents. Scale bar: 50 µm. **c** Cytotoxic effects were further excluded by analysis of LDH secretion in the undernatants of the organoids at day 12 of airlift compared to untreated samples. Statistics: one-way ANOVA with Dunett’s T3 post hoc test; *p* ≤ 0.05 *, *p* ≤ 0.01 **, *p* ≤ 0.001 *** compared to untreated PBS control, *n* = 6 skin equivalents measured in technical duplicates.

Topical treatment approaches were performed using the glucocorticoid dexamethasone (DEX) or the natural compound celastrol (CEL) derived from *Tripterygium wilfordii* in a preventive manner. For this, the compounds were applied on day 0, 6 and 12 of airlift incubation (Fig. 4a). HE staining of the treated skin models revealed similar morphological appearances under treatment compared to PBS-treated control samples. The PSO-HSE showed signs of parakeratosis (retention of nuclei in the *stratum corneum*) which was also present after DEX or CEL treatment (Fig. 4b). A concentration of 10 µM DEX did not increase the LDH release neither in the physiological skin equivalents nor in the PSO-HSE. CEL slightly decreased the LDH secretion of the physiological skin models. Under PSO conditions, the LDH release was significantly higher after CEL treatment but still comparable to levels secreted by PBS treated substitutes (Fig. 4c). The vehicle control DMSO did not affect the LDH release (Supplementary Fig. 1a).

### Anti-inflammatory and protein regenerative properties of topical treatment approaches

**Fig. 5:**
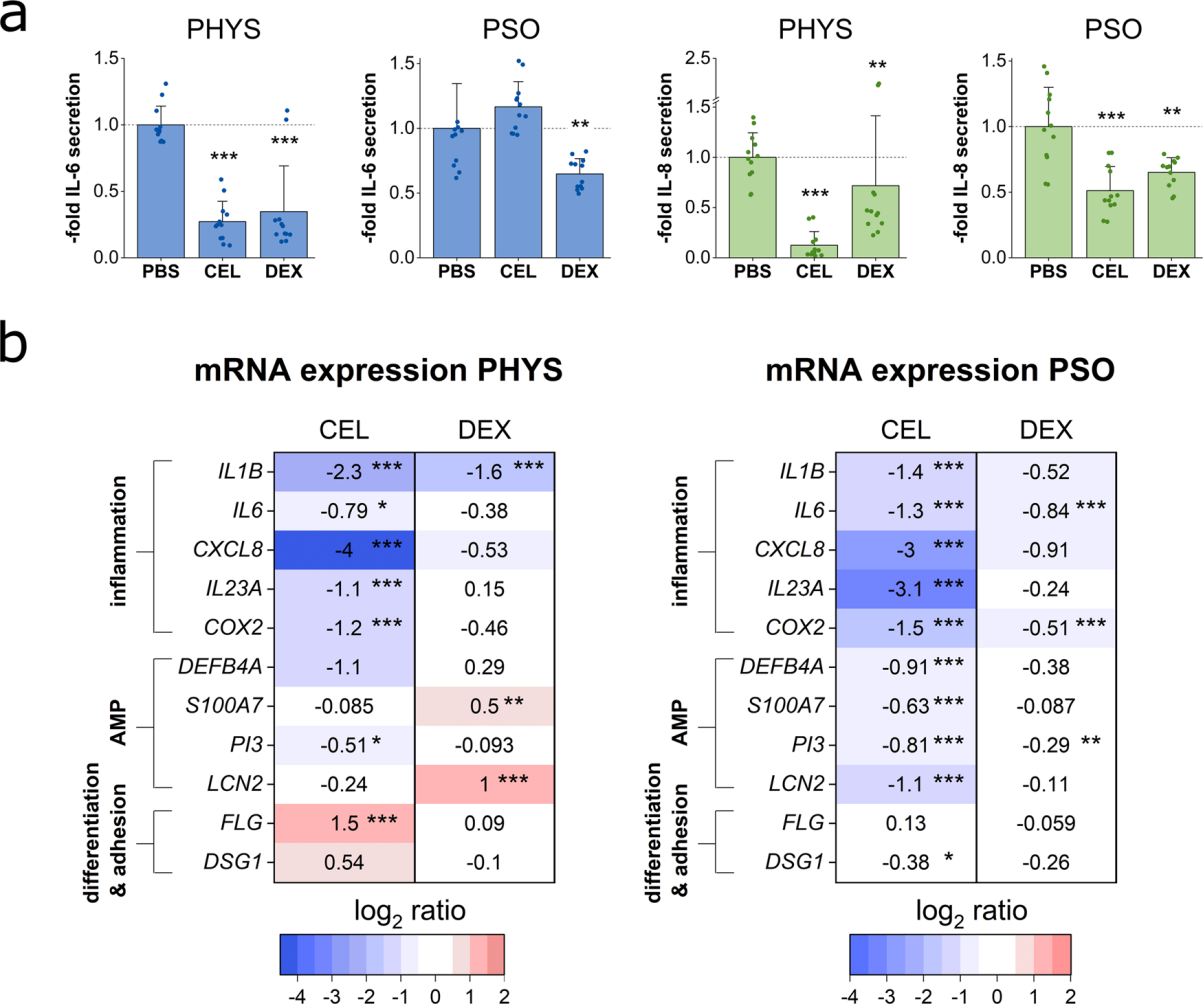
Topical treatment effects on cytokine secretion and gene expression. 3D skin equivalents were cytokine-primed for PSO induction and celastrol (CEL) or dexamethasone (DEX) were applied topically at day 0, 6 and 12 of airlift incubation. (**a**) Protein secretions of IL-6 (blue) and IL-8 (green) in the undernatants of the organoids at day 12 were measured by ELISA to investigate anti-inflammatory properties of the topical therapies. Statistics: Mann–Whitney *U* test; *p* *≤* 0.05 *p *≤* 0.01 **p *≤* 0.001 *** compared to untreated PBS control, *n* *=* 6 skin equivalents measured in technical duplicates. **b** Gene expression was analysed via qPCR. The mRNA expression of genes altered in PSO milieu was investigated to examine reverting effects under topical treatment. Genes of interest include genes encoding inflammatory markers like interleukin 1β (IL1B), interleukin 6 (IL6), chemokine (CXC motif) ligand 8 (CXCL8), interleukin 23 A (IL23A) and cyclooxygenase-2 (COX2), genes encoding antimicrobial peptides (AMP) like beta-defensin-2 (DEFB4A), psoriasin (S100A7), elafin (PI3) and lipocalin-2 (LCN2) and genes encoding the differentiation marker filaggrin (FLG) or the desmosomal cell-cell-contact protein desmoglein-1 (DSG1). Expression values relative to the untreated PBS control were log_2_ transformed. This transformation leads to a mean log_2_ value *=* 0 for PBS control. Upregulations of mRNA levels upon treatment are shown in red for values *>* 0 while downregulations are depicted in blue for values *<* 0. Statistics: Mann–Whitney *U* test; *p* *≤* 0.05 *, *p* *≤* 0.01 **, *p* *≤* 0.001 *** compared to untreated PBS control, *n* *=* 5 skin equivalents measured in technical duplicates.

Anti-inflammatory effects on the release of the pro-inflammatory cytokines IL-6 and IL-8 were investigated by ELISA (Fig. 5a). CEL and DEX potently decreased the IL-6 secretion of the physiological skin models. However, after treatment of the PSO-HSE, only DEX reduced the IL-6 secretion while CEL did not result in an inhibition. CEL and DEX treatment resulted in significantly lower levels of IL-8 secretion under physiological as well as disease-associated conditions. The vehicle control DMSO did not affect the secretion of IL-6 and IL-8 (Supplementary Fig. 1b). To assess regenerative effects on the pathologically altered protein expressions, IHC was performed. However, topical treatment with 10 µM DEX or with 10 µM CEL did not revert the low CK10 and FLG levels of the PSO-HSE. Furthermore, under topical therapy, the hyperproliferative phenotype with a high number of Ki67^+^ basal cells remained in the PSO-HSE. The inflammatory CK16 still appeared in high levels after treatment with DEX. However, lower levels were observed after CEL treatment in the PSO-HSE (Supplementary Fig. 2).

### Transcriptional modulation by topical treatment approaches

Gene expression analyses were carried out to detect putative counter regulatory effects on mRNA transcripts after topical treatment of the skin equivalents. CEL application to physiological skin models significantly reduced the expression of genes encoding inflammatory mediators like *IL1B*, *IL6*, *CXCL8*, *IL23A* and *COX2*. DEX significantly inhibited the *IL1B* expression while other inflammation-associated genes were only marginally repressed. The mRNA expressions of the AMP genes *DEFB4A* and *PI3* were downregulated upon CEL treatment. DEX, in contrast, boosted the gene expression of AMPs like *S100A7* and *LCN2*. The *FLG* gene was significantly upregulated by CEL but remained unaffected upon DEX administration.

In the PSO-HSE, CEL treatment significantly downregulated the gene expression of the inflammation-associated genes *IL1B*, *IL6*, *CXCL8*, *IL23A* and *COX2*. DEX led to a significant reduction in the gene expression of *IL6* and *COX2*, while *CXCL8* and *IL1B* were only tending to be downregulated under PSO conditions. The glucocorticoid did not affect the mRNA levels of *IL23A*. A significant decrease of the high psoriatic expression levels of the AMP genes *DEFB4A*, *S100A7*, *PI3* and *LCN2* was observed upon CEL treatment. DEX did not distinctly modulate the AMP expression of the PSO-HSE. Only the psoriatic mRNA levels of *PI3* were reduced by DEX treatment, however, a similar reduction was noted for the vehicle control (Supplementary Fig. 1c). Genes like *FLG* encoding the structural component filaggrin or *DSG1* encoding the desmosomal adhesion molecule desmoglein-1 were not upregulated by the topical therapies of the PSO-HSE. (Fig. 5b).

### Skin compatibility of subcutaneous treatment with antibody biologics

**Fig. 6:**
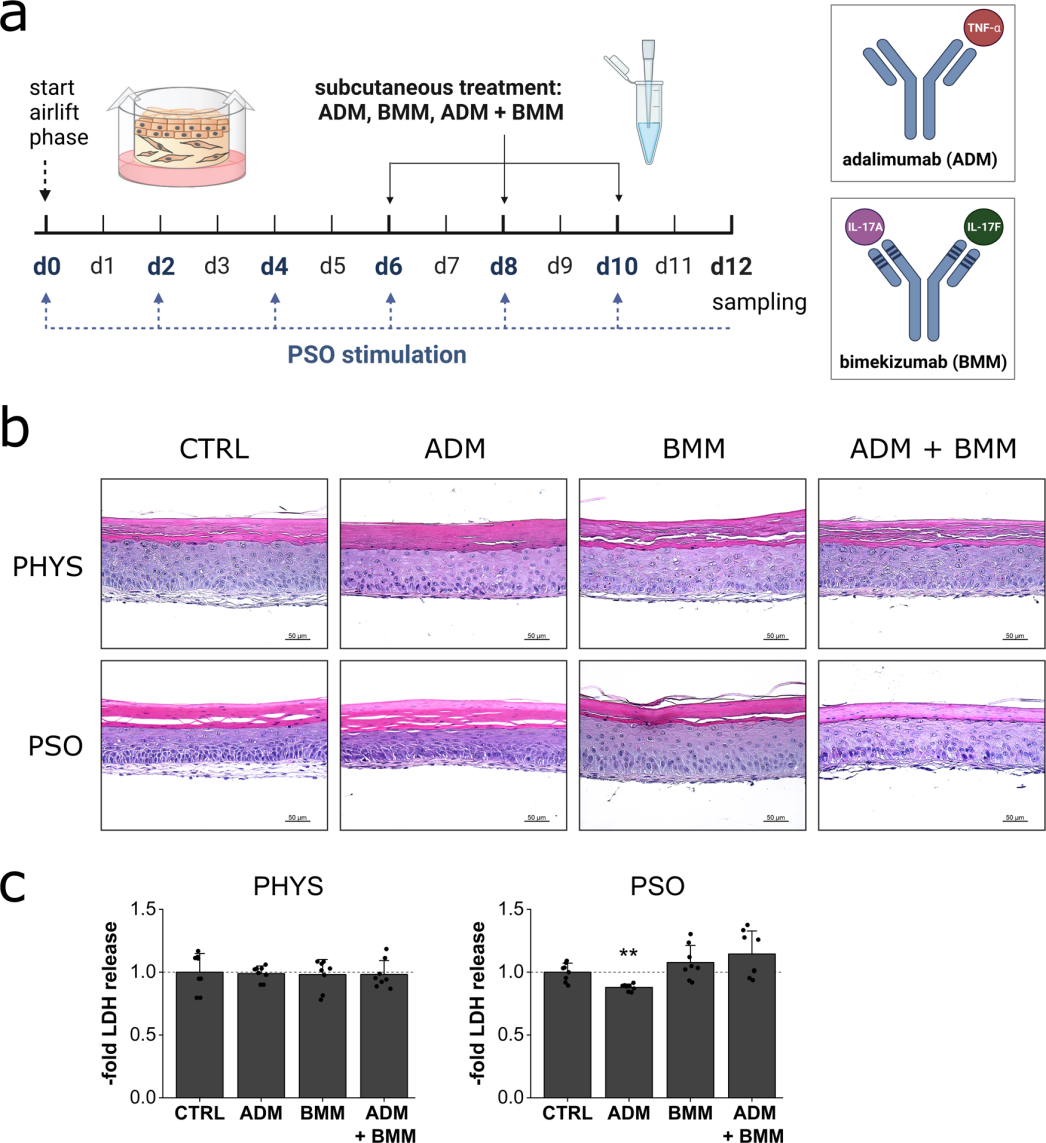
In vitro skin compatibility of subcutaneously applied biologics for treatment of PSO skin equivalents. **a** 3D skin equivalents were cultivated physiologically (PHYS) or cytokine-primed for psoriasis (PSO) induction. Adalimumab (ADM), bimekizumab (BMM) or a combination of ADM + BMM were applied subcutaneously at day 6, 8 and 10 of airlift incubation. Blue arrows indicate the time points of the medium exchange and priming with the cytokine cocktail while the dotted line represents the presence of the cytokines in the medium reservoir. Created in BioRender. Reddersen, K. (2024) https://BioRender.com/c34s364. **b** HE staining was used to reveal putative cytotoxic effects via morphological changes after treatment of PHYS or PSO equivalents. Scale bar: 50 µm, representative pictures of *n* = 2 skin equivalents. **c** Cytotoxic effects were further excluded by analysis of LDH secretion in the undernatants of the organoids at day 12 compared to untreated samples. Statistics: one-way ANOVA with Dunett’s T3 post hoc test; *p* ≤ 0.05 *, *p* ≤ 0.01 **, *p* ≤ 0.001 *** compared to untreated control, *n* = 4 skin equivalents measured in technical duplicates.

Subcutaneous biological therapy with antibodies selectively targeting cytokine PSO mediators involved in the disease pathology was investigated. Here, the antibodies adalimumab (ADM) targeting TNF-α and bimekizumab blocking IL-17A/F were tested. PSO induction was mediated by cytokine supplementation and medium exchange every other day. Concomitantly, the biological antibodies were applied in the subcutaneous medium reservoir at day 6, 8 and 10 of airlift incubation (Fig. 6a). HE stained physiological (PHYS) skin substitutes as well as PSO-HSE showed no morphological changes compared to untreated controls. Parakeratosis of the PSO-HSE was still observable under combination therapy with ADM + BMM (Fig. 6b). The antibodies did not alter the LDH release when applied to physiological skin models. In PSO-HSE, the antibody therapy was likewise well tolerated without significantly inducing the LDH secretion (Fig. 6c).

### Anti-inflammatory and protein regenerative properties of subcutaneous treatment with antibody biologics

**Fig. 7:**
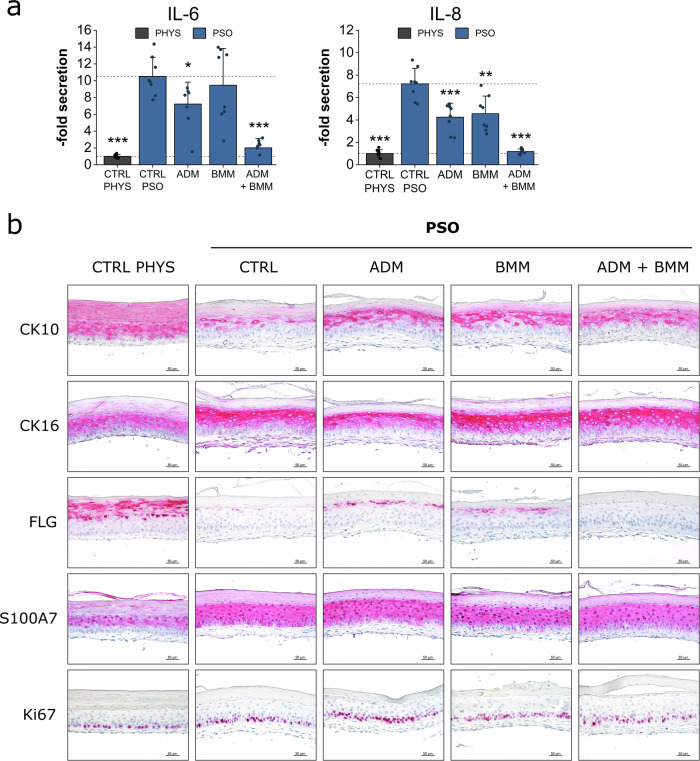
Biologics treatment effects on cytokine secretion and protein expression. 3D skin equivalents were cultivated physiologically (PHYS) or cytokine-primed for psoriasis (PSO) induction. Adalimumab (ADM), bimekizumab (BMM) or a combination of ADM + BMM were applied subcutaneously at day 6, 8 and 10 of airlift incubation. **a** Protein secretions of IL-6 and IL-8 in the undernatants of the organoids at day 12 were measured by ELISA to investigate anti-inflammatory properties of the antibody therapies. Statistics: Mann–Whitney *U* test; *p* ≤ 0.05 *, *p* ≤ 0.01 **, *p* ≤ 0.001 *** compared to untreated PSO control, *n* = 4 skin equivalents measured in technical duplicates. **b** Effects on protein production of the differentiation markers keratin-10 (CK10), filaggrin (FLG), pathological keratin-16 (CK16), the antimicrobial peptide psoriasin (S100A7) and the mitosis marker Ki67 were analysed by IHC. The protein of interest is stained in red by IHC processing. Scale bar: 50 µm, representative pictures of *n* = 2 skin equivalents.

Anti-inflammatory effects on the release of the pro-inflammatory cytokines IL-6 and IL-8 were investigated by ELISA (Fig. 7a). Untreated PSO-HSE secreted high levels of IL-6 and IL-8. ADM significantly reduced the secretion of both, IL-6 and IL-8, while BMM only decreased the release of IL-8. The combination therapy of both biologics resulted in a more effective reduction of both cytokines compared to single antibody treatments. Upon ADM + BMM therapy of the PSO-HSE, the release of IL-6 and IL-8 was nearly reduced to levels similar to physiological controls (Fig. 7a). To assess regenerative effects on the pathologically altered protein expressions, immunohistochemistry (IHC) was performed. Treatment with ADM, BMM or a combination of both induced the re-production of CK10 compared to the low protein levels observed in the untreated PSO control. In contrast, the pathologically increased levels of CK16 remained after biological therapy. FLG was not reverted to physiological levels after application of the antibodies. However, a slight induction was observed under single antibody treatment but not under combination therapy. The high psoriatic protein expression of the AMP S100A7 and the proliferation marker Ki67 persisted upon the biological therapies during the observation period (Fig. 7b).

### Transcriptional modulation by subcutaneous treatment with biologics

**Fig. 8:**
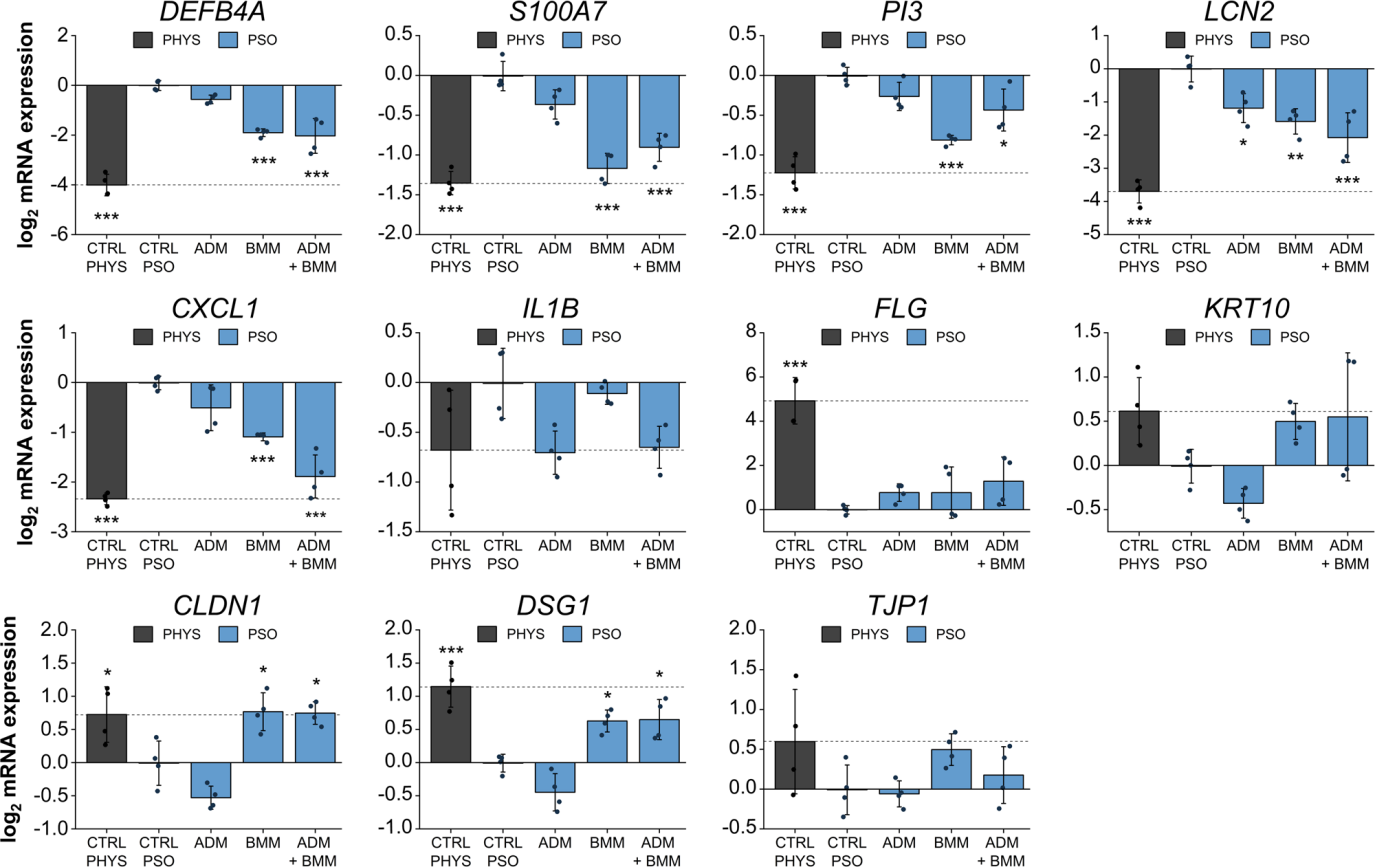
Biologics treatment effects on gene expression. 3D skin equivalents were cultivated physiologically (PHYS) or cytokine-primed for psoriasis (PSO) induction. Adalimumab (ADM), bimekizumab (BMM) or a combination of ADM + BMM were applied subcutaneously at day 6, 8 and 10 of airlift incubation. Gene expression was analysed via qPCR. The mRNA expressions of genes altered in PSO milieu (blue) were investigated to examine reverting effects under biological treatment in relation to physiological gene expression (grey). Genes of interest include genes encoding antimicrobial peptides (AMP) like beta-defensin-2 (*DEFB4A*), psoriasin (*S100A7*), elafin (*PI3*) and lipocalin-2 (*LCN2*), genes encoding inflammatory markers like chemokine (CXC motif) ligand 1 (*CXCL1*) and interleukin 1β (*IL1B*), genes encoding the differentiation markers filaggrin (*FLG*) and keratin-10 (*KRT10*) or genes encoding cell-cell contact proteins like claudin-1 (*CLDN1*), desmoglein-1 (*DSG1*) and tight junction protein-1 (*TJP1*). Expression values relative to the untreated PSO control were log_2_ transformed. This transformation leads to a mean log_2_ value = 0 for the untreated PSO control. Upregulations of mRNA levels upon treatment are indicated by values > 0 while downregulations are indicated by values < 0. Statistics: one-way ANOVA with Bonferroni post hoc test; *p* ≤ 0.05 *, *p* ≤ 0.01 **, *p* ≤ 0.001 *** compared to untreated PSO control, *n* = 2 skin equivalents measured in technical duplicates.

Gene expression analyses were carried out to detect putative counter regulatory effects on mRNA transcripts after biological treatment of PSO-HSE. ADM treatment resulted in a slight downregulation of the AMP genes *DEFB4A*, *S100A7*, *PI3* and significant reduction of *LCN2* expression. A more pronounced reduction of AMP mRNA levels was observed after BMM therapy. Upon BMM treatment, the gene expression of *S100A7* and *PI3* even nearly reverted to physiological transcript levels. A similar inhibition of the AMP gene expression of *DEFB4A*, *S100A7*, *PI3* and *LCN2* was found after ADM + BMM combination treatment. ADM only slightly reduced the *CXCL1* gene expression while treatment with BMM potently downregulated the chemokine gene. An additive effect of the *CXCL1* downregulation was observed under combination treatment of ADM + BMM. *IL1B* was downregulated upon ADM alone or in combination with BMM resulting in normalized transcript levels of this cytokine gene. None of the antibody treatments did increase the *FLG* gene expression to a physiological relevant level, still, initial inductions were recorded. TNF-α blockage via ADM did not lead to an upregulation of the *KRT10*, *CLDN1*, *DSG1* and *TJP1* gene expression. In contrast, the mRNA levels of *KRT10*, *CLDN1* and *DSG1* were even lower than in the untreated PSO control. BMM resulted in elevated levels of *KRT10* and *CLDN1* transcripts comparable to physiological levels. This result also occurred after treatment with BMM in combination with ADM where the effect is likely attributed to the blockage of IL-17. In addition, significantly higher mRNA expressions were measured for *DSG1* and by trend for *TJP1* upon BMM therapy (Fig. 8).

## Discussion

Using in vitro models as preclinical tools for therapeutic testing requires the knowledge of their responsiveness range. The lifespan of in vitro cultured skin models is limited^44^. Collagen-based skin equivalents are prone to contraction with a narrow culture shelf life of these artificial tissues^45–47^. A lifetime prolongation of cutaneous in vitro models can be achieved by defining culture conditions or replacing the contractile animal collagen^48–50^. The finite lifetime of these skin models indicates a limited time frame for pharmacodynamic use. Thus, it is reasonable to pre-evaluate their response potential and recovery ability to overcome pathological changes. In addition, it is essential to predict and contextualize, which pathological attributes might persist under treatment and/or require prolonged treatment durations. After 6 days of PSO priming, the pathological stimulus was removed and the skin models were cultivated for another 6 days for regeneration. Within the chosen observation period, the skin models were able to re-synthesize structural proteins like CK10 and FLG to re-establish the cutaneous integrity. This observation points out, that the skin models are not inferior to a long-term skin model for PSO described by Singh et al. in which a FLG recovery after biological therapy was observed^50^. The downregulated expression of the *FLG* and *KRT10* gene was increased after 6 days of regeneration in contrast to the PSO_12 d_-HSE. Compared to the physiological expression, the mRNA levels of *FLG* and *KRT10* were still lower in the regenerated PSO-HSE. Nevertheless, the significant increase in mRNA expression can be considered an adequate response since the protein levels of FLG and CK10 were completely restored. It can be assumed that the increase in mRNA expression was efficiently translated to protein levels. A similar phenomenon was observed by Lee et al., who treated atopic dermatitis skin models with a licorice-derived compound. As revealed by IHC staining, the FLG protein expression was nearly completely restored after treatment with the highest compound concentration, while the mRNA levels were increased but still strikingly lower than the healthy control^51^. Hence, crossing a certain threshold of mRNA expression seems to be abundant for restoration of the FLG protein production. Apart from structural differentiation markers, cellular adhesion was normalized after the short regeneration period as well. All tested cell-cell contact genes, namely *CLDN1*, *TJP1* and *DSG1* have been upregulated towards physiological expression levels.

Interestingly, persistent pathologies were determined with remaining high levels of S100A7 protein levels and a continuous hyperproliferatory Ki67 signature after 6 days of regeneration. While impaired differentiation processes seem to revert rapidly, the activated host defence via AMP overexpression as well as the proliferatory dysregulation likely represent attributes with a decelerated treatment response. These findings correlate with in vivo patient conditions. For instance, 5-7 days of calcipotriol treatment reduced the S100A7 protein expression in the skin of PSO patients but the levels were still higher compared to healthy skin^52^. Similar observations have been reported for Ki67 in psoriatic skin. The proliferation marker was only slightly reduced after day 14 of treatment while complete normalization was recorded for day 28 indicating a gradually but decelerated recovery over time^53^. In addition, patient studies usually examine therapeutic effects after months of treatment^54–56^. This underlines the rather time-consuming healing phase of psoriatic plaques. To be precise, newer findings indicate that the treatment response of patients might align with a so-called ‘hit hard and early’ approach. Patients benefit from an early intervention, which maximizes treatment outcomes and prevents early remission^57^. In chronic stages, tissue-resident memory t-cells (TRM) residing in the epidermis develop an immunological memory and seem to play a crucial role in disease relapse, thus, hampering the treatment response^58^. The elevated S100A7 and Ki67 protein levels within the in vitro tissues after 6 days of regeneration reflects the in vivo nature of psoriatic lesions. Nevertheless, the initiation of AMP suppression was observed on mRNA levels. After 6 days without the PSO stimulus, all tested AMP genes, namely *DEFB4A*, *S100A7*, *PI3* and *LCN2*, completely reverted to physiological expression levels. Thus, it can be assumed that the recovery of AMP protein levels will follow up and can be regarded a matter of time. In addition, all inflammation-associated genes (*IL1B*, *IL6*, *CXCL8*, *CXCL1*, *COX2* and *IL23A*) showed a resolution towards physiological gene expression values. The release of the inflammatory mediators IL-6 and IL-8 was also entirely reversed. This confirms a swift normalization of inflammatory keratinocyte reactions as soon as the pathological stimulus is removed. Not only do IL-6 and IL-8 reflect the disease severity in PSO patients but they also correlate with treatment responses^59–61^. Since, both interleukins were effectively normalized by exposing the PSO-HSE to physiological conditions, their usage as predictive biomarkers for treatment has been confirmed. This underlines their analogy to in vivo skin. In summary, differentiation processes were normalized, the inflammatory phenotype was alleviated and the regenerative effects covered all tested genes for inflammation, differentiation, AMP expression and cell-cell-contacts. However, proteinary alterations like the elevated AMP production and hyperproliferative signals were revealed as late responding treatment parameters. In conclusion, the present in vitro models of PSO are able to recover from the pathological condition. Thus, they mimic an in vivo-like response behaviour qualifying them as preclinical tools for treatment response studies.

After confirming the responsiveness, the in vitro models were used to conduct treatment experiments. First, a topical therapy approach was executed. As topical reagents, the glucocorticoid dexamethasone (DEX) or the natural compound celastrol (CEL) were applied. DEX is a potent synthetic glucocorticoid suppressing immune reactions and inflammation processes^62^. Glucocorticoids have a solid history in the treatment of inflammatory conditions like (auto-)immune diseases, asthma or inflammatory bowel disease^63^. Hence, DEX represents an ideal candidate to test its efficacy in inflammation-associated 3D skin models of PSO. CEL, on the other hand, is a natural compound that can be isolated from *Tripterygium wilfordii hook*, which was used as an effective ingredient in traditional Chinese medicine to treat inflammatory disorders including PSO^64^. However, due to cytotoxic side effects, its usage is no longer recommended^65^. CEL has been identified as one of the active compounds of this Asian plant and anti-inflammatory and anti-proliferative properties haven been described^22–26^. In addition, the compound was proven to exert anti-psoriatic effects^27,28^. Anti-tumoral properties of the compound have been associated with its cytotoxicity dependent on the concentration^66,67^. In our study, neither 10 µM DEX nor 10 µM CEL did induce pathological alterations of the microanatomy. In accordance, the LDH release was not elevated following topical applications of DEX or CEL. A weakened cutaneous barrier has previously been shown for the PSO-HSE^32^. This might lead to increased compound penetration and thus higher susceptibility to cytotoxic events. Compound concentrations of 10 µM were well tolerated even when applied to PSO-HSE with barrier defects. In contrast, cells in monolayer have been shown to be more prone to cytotoxic effects of lower CEL concentrations. Zhou et al. identified IC_50_ values of 1.1 µM CEL on HaCaT keratinocytes or 2.9 µM on primary human keratinocytes (HEK)^68^. A viability reduction of fibroblasts exposed to CEL has also been reported for concentrations ≥ 2 µM^69^. As for DEX, a concentration of 1 µM of the corticosteroid was found to be cytotoxic in cell monolayers of osteoblasts^70–72^. HaCaT keratinocytes, in contrast, were proven to tolerate 1 µM DEX while their cell viability was decreased by a concentration of 10 µM DEX^73^. This verifies 3D skin models to be more suitable for drug testing than simple monolayer cultures. A study by Sun et al. confirmed the ability of cutaneous cells to be more resistant against oxidative stress and toxic compounds in an 3D environment compared to 2D conditions^74^. By comparison of HaCaT cells and 3D skin equivalents, it has been demonstrated that the 3D skin models were not irritated by compound concentrations which exerted cytotoxic effects in the monolayer culture. In addition, the pharmacological compatibility of these skin models was similar to in vivo skin^75^. This supports the general acceptance of 3D skin models to mimic in vivo-like conditions and predict skin irritative aspects. Due to the 3D microanatomy and the development of a horny layer, higher drug concentrations can be tolerated compared to vulnerable 2D monolayer.

The topical therapies with CEL or DEX exerted anti-inflammatory effects under psoriatic as well as physiological conditions. The secretion of the pro-inflammatory mediators IL-6 and IL-8 was significantly reduced due to CEL or DEX. But as an exception, topical CEL treatment failed to decrease the IL-6 secretion of PSO-HSE. Here, the stimulation with the PSO cytokine mix seems to dominate the effect on the IL-6 release outplaying the anti-inflammatory reach of the natural compound. CEL was shown to downregulate the IL-6 release via inhibition of the NF-κB signalling cascade^76^. On the other hand, elevated levels of the activated phosphorylated NF-κB form induced by TNF-α have been described for psoriatic lesions^77^. Since the PSO stimulation mix contained TNF-α, an activation of the NF-κB pathway in the cells of the PSO-HSE seems very likely. Due to modulation of the same signalling pathway by CEL and TNF-α, competitive interactions can be assumed. In contrast to protein secretion, the mRNA expression of *IL6* was decreased after CEL treatment of the PSO models. Further, inflammation-associated genes like *IL1B*. *CXCL8*, *IL23A* and *COX2* were downregulated in the PSO-HSE due to CEL demonstrating the anti-inflammatory impact of the compound. A CEL-induced repression of these genes was also observed after treatment of PHYS samples. These transcriptomic modulations are in accordance with downregulated mRNA levels of *IL1B*, *IL6*, *CXCL8* and *COX2* found after CEL treatment in vitro and in vivo^28,69,76,78,79^. Interestingly, CEL led to a more potent inflammatory gene repression than the glucocorticoid DEX. CEL has a glucocorticoid-like structure which led to the former hypothesis that celastrol’s mode of action involves glucocorticoid receptor (GR) signalling. However, challenging the natural compound with a potent GR antagonist did not alter its effect on cytokine secretion^80^. This indicates that DEX and CEL repress inflammatory events in a different manner, which can explain the differences observed in this study. Especially *CXCL8* was significantly reduced under CEL treatment in PSO samples, while DEX only reduced its gene expression without reaching statistical significance. Similar results were found in epithelial cells, where DEX preconditioning reduced TNF-α-mediated induction of the *CXCL8* mRNA expression only by trend^81^. This indicates a competitive interference of TNF-α-associated induction versus corticosteroid-mediated repression. Despite different effects of CEL and DEX on transcript levels of the IL-8 encoding gene *CXCL8*, the protein secretion was significantly lowered by both compounds. This underlines their anti-inflammatory potential and the effectiveness in PSO treatment. IL-8 is a cytokine that is highly produced in the psoriatic skin driving keratinocyte overgrowth and angiogenesis^82–84^. DEX not only decreased IL-8 but also IL-6 secretions of the inflammatory in vitro model of PSO. These results are consistent with a DEX-mediated reduction of both cytokines measured in another inflammation model of a skin organ culture pre-stimulated with LPS^85^. A significant reduction of the inflammation-associated genes *IL6* and *COX2* was observed after DEX treatment of PSO-HSE. These results are consistent with other in vitro findings of DEX downregulating the mRNA levels of inflammatory genes like *IL6*, *COX2* and *IL1B* in other cell types^81,86^. The *IL6* expression can be regulated by corticosteroids like DEX via GR binding sites in the promotor region of this gene^87^. In addition, DEX was found to destabilize the mRNA stability of *COX2*^88^.

Interestingly, CEL and DEX showed rather different effects on AMP gene expression. Under physiological conditions, DEX upregulated AMP genes like *S100A7* and *LCN2* while AMP transcripts remained unaffected or in case of *PI3* rather downregulated under CEL. In PSO-HSE, CEL potently downregulated all tested AMP genes whereas DEX had almost no effect on *DEFB4A*, *S100A7* and *LCN2*. The observed effect of CEL on AMP mRNA levels is consistent with the findings of Nguyen et al., who measured lower gene expression values of *DEFB4A*, *PI3* and *S100A7* in PSO-primed NHEK due to CEL pre-incubation^78^. A reduction of the elevated AMP production in PSO patients is an advantageous treatment aim. AMP function as host defence peptides targeting pathogens that were able to invade due to wounding or weakened barrier conditions. The high AMP levels explain the lower number of bacterial or viral infections found in PSO patients compared to those suffering from atopic dermatitis^89,90^. However, AMPs do not only participate in immune reactions but also lead to an enhanced recognition of danger-associated molecular patterns (DAMPs). DAMPs like self-DNA or RNA often occur as a result of scratching^4^. Inflammation induced by DAMPs has been linked to the Koebner phenomenon, a reaction with manifestations of new lesions on previously unaffected skin after cutaneous injury ^91^. Unlike CEL, DEX upregulated the *LCN2* gene under physiological conditions. In the PSO-HSE, the glucocorticoid did not further induce transcripts of this AMP gene since its expression is already at high levels due to the psoriatic stimulation. The finding of the *LCN2* upregulation is in accordance with other in vitro studies, which confirmed the AMP-inducing effect of DEX on gene expression of *LCN2* and genes encoding beta-defensins^92,93^. The upregulation of *LCN2* due to DEX aligns with the presence of a glucocorticoid response element (GRE) in the promotor region of this gene^92^. In accordance, CEL could be the first choice over DEX for PSO treatment due to its AMP-decreasing features.

Recovery effects of structural proteins and differentiation markers like CK10 and FLG upon topical treatment were not observed after the short-term therapy. Another in vitro study provided the statement of an FLG induction due to preventive pre-treatment with a CEL-containing extract. However, the effect was only slightly significant compared to untreated samples and still almost 50% lower than the healthy control^78^. In the present study, CEL did not qualitatively increase the pathological low FLG protein levels and the compound did not induce the *FLG* mRNA expression in the PSO-HSE. However, CEL led to an upregulation of *FLG* in PHYS skin equivalents. This supports the finding of a filaggrin-inducing effect by CEL. The phenomenon of the missing FLG induction in the PSO models might again be explained by competitive interferences of the cytokine cocktail and the natural compound. CEL is known to inhibit the signal transducer and activator of transcription-3 (STAT3)^94^. STAT3 is involved in differentiation regulation. Inhibition of the transcription factor leads to an increased filaggrin production^95^. This might provide a putative mechanism by which CEL leads to an FLG induction. Psoriatic lesions have been characterized by increased expression and activation of STAT3 induced by PSO-associated cytokines like IL-22 and IL-6^96,97^. With this, a competitive modulation of the STAT3 signalling can be assumed in the PSO-HSE. Here, the effect of the PSO cytokines on STAT3 seems to prevail over the CEL-mediated regulation. DEX treatment did not induce the *FLG* expression. This result is consistent with a patient study of atopic dermatitis, another inflammatory skin disease, where the effects on *FLG* gene expression after topical treatment with a glucocorticoid were considered minimal^98^. In addition, the *DSG1* mRNA expression was not significantly induced upon the topical therapies indicating non-sufficient effects on the reversion of cellular adhesion. The number of Ki67^+^ cells still appeared to be elevated in the PSO-HSE despite multiple applications of the topical compounds. Several in vitro studies with cellular monolayer postulate anti-proliferative effects of CS^25,68,99^. In a PSO mouse model, CEL reduced the hyperproliferative Ki67^+^ signal in the basal layer^100^. The experimental setup with daily application of CEL for 6 days differed from the procedure of the present study. Hence, it might be possible that the triple treatment procedure distributed over 12 days was not enough for CEL to provide anti-mitogenic actions overcoming the hyperproliferative phenotype of the PSO models. Another influencing parameter is the formulation of the applied compound. Qiu et al. have demonstrated the impact of various CEL formulations on proliferation inhibition^100^. In this study, CEL was dissolved in DMSO and further diluted in PBS resulting in a remaining DMSO fraction of 0.2%. The used preparation of the lipophilic substance might be less effective in terms of tissue penetration. In addition, the hyperproliferatory phenotype was pre-evaluated as a tenaciously recovering feature of the in vitro skin models that even persisted after 6 days without the PSO stimulation.

In summary, the topical therapies alleviated the inflammatory signature of the disease-associated skin models. However, other pathological alterations were not potently reverted, indicating persistent disease features. Under in vivo conditions, topical therapies with anti-inflammatory compounds do not only act on cutaneous cells but also inhibit the inflammatory signalling of immune cells. In this in vitro model the therapeutic range is limited to fibroblasts and keratinocytes and even impeded by the continuous stimulation with the cytokine mix. This might be a limitation of the present model. On the other hand, anti-psoriatic activities were detected upon the topical therapies with CEL or DEX underlining their anti-inflammatory effectiveness even under continuous cytokine stimulation mimicking activated Th lymphocytes.

To investigate the response of the in vitro models to systemic therapies, biological drugs like adalimumab and/or bimekizumab were applied subcutaneously. Morphological examinations and monitoring of the LDH release confirmed the in vitro compatibility of the antibody drugs that have already been in clinical use. Their clinical safety has been assessed and reported by the European Medicines Agency ^20,101^. Interestingly, controverse research results have been discussed regarding the cytotoxicity of TNF-α blockers like ADM. In vitro studies reported an activation of the complement system after antibody binding of transmembrane TNF-α leading to a complement-dependent cytotoxicity^102,103^. However, others did not detect complement-dependent or other cytotoxic events after ADM administration^104,105^. Our findings of no cytotoxic effects after triple application of the biologicals within the final 6 days of airlift incubation are in accordance with the latter reports and align with the confirmed safety statements required for their clinical approval.

After biological therapy of the PSO-HSE, recuperating effects have been demonstrated on protein levels. Mono- as well as dual therapy with ADM and BMM resulted in increased protein expression of CK10 compared to low levels seen in untreated PSO samples. This might account for the improved PASI scores observed in PSO patients after receiving ADM or BMM treatment^106–108^. Proteomic changes involving several molecular pathways have been identified after biological blockage of TNF-α or IL-17. Among the most significantly changed proteins, keratin-2 was named as a hit, which has been linked to a PASI improvement^109^. This supports the suggestion of positive effects on keratin proteins due to treatment with the biologicals tested in this study. Interestingly, ADM did not induce the *KRT10* mRNA expression indicating a protein modulatory mechanism or the involvement of another interaction partner inducing or stabilizing the CK10 protein expression. In contrast, BMM increased the *KRT10* gene expression comparable to physiological levels. In vivo blocking of the IL-17A receptor confirms a normalization of PSO-decreased *KRT10* mRNA levels^110^. Consequently, IL-17 seems to be a key cytokine reducing the *KRT10* gene expression. Pfaff et al. confirmed this assumption by measuring lower levels of *KRT10* transcripts in 3D skin models upon IL-17 stimulation^111^. In addition, Singh et al. have shown increasing levels of *KRT10* mRNA levels after treatment of IL-17A-stimulated skin models with an IL-17A antagonist supporting the results of the present study ^50^. The pathologically elevated protein expression of the inflammatory CK16, however, was not reverted upon the antibody therapies. A clinical study has shown time-dependent effects on the CK16 protein expression upon therapy with a TNF-α inhibitor. After 4 weeks, a CK16 reduction was noted. However, the levels still appeared to be distinctly higher than in uninvolved skin. A nearly complete clearance and normalization was observed after week 12 of treatment^112^. Similar results of a CK16 resolution to a physiological baseline level after weeks have been shown for anti-IL-17 therapies^113,114^. It can be assumed that certain pathological alterations due to PSO dysregulations cannot be overcome after a few days of treatment but weeks instead. In addition, ADM and BMM solely inhibit TNF-α and IL-17A while other mediators of the PSO stimulation mix, especially IL-22, continue with stimulating cutaneous cells. IL-22 has been identified as potent inducer of the CK16 production^115,116^. Consequently, IL-22 might sustain the CK16 levels in the present PSO in vitro model even under TNF-α and/or IL-17 inhibition. FLG protein levels were also not potently increased under the biological approach. Patient treatment with a TNF-α inhibitor resulted in restored FLG levels after 12 weeks^117^. Blocking of TNF-α via ADM or IL-17A/F via BMM did not significantly induce the FLG mRNA expression which might be causative for the absent regeneration of the FLG protein production observed in the present study. In contrast, Singh et al. stated a restored FLG protein expression and increasing *FLG* mRNA levels after anti-IL-17 treatment of their IL-17A-primed skin models^50^. However, their experimental setting differs from the procedure of this study. Singh et al. solely primed their skin models for 5 days with IL-17A and treated them with or without continuing IL-17A stimulation with an IL-17A blocker for 14 days. In the present study, a shortened treatment period of 6 days was conducted. In addition, a stimulation mix containing multiple cytokines (IL-17A, IL-22, IL-6, IL-1α, TNF-α) was used. PSO is a multifactorial disorder that includes a dysregulation of various cytokines^41^. Therefore, priming skin equivalents with various cytokines for PSO induction seems to be closer to in vivo conditions. Despite blockage of TNF-α or IL-17, IL-22 is still present in our setting. This cytokine is an essential suppressor of the filaggrin mRNA and protein expression^118–120^. A filaggrin normalization can be achieved when the pathological signalling is completely removed as shown by the pre-evaluating response tests. Remaining cytokines that were not blocked by the biological antibodies thus hampered a FLG/*FLG* recovery underlining the crucial impact of these inflammatory molecules and their pathological interplay in PSO progression.

Basal hyperproliferation was observed in the untreated PSO-HSE. The number of Ki67^+^ cells remained elevated after biological therapy compared to the physiological control. Hence, hyperproliferation might be another slow-responding parameter as confirmed by the initial regeneration experiment. After 12 weeks of anti-IL-17 or anti-TNF-α therapies, the Ki67 signals completely reverted in the skin of PSO patients^112,113^. Studies with regular patient monitoring have shown Ki67-reducing effects of the IL-17 antagonist secukinumab after 1 or 2 weeks respectively. However, the hyperproliferative signal was still highly increased over the non-lesional baseline level^113,121^. Similar results have been described for etanercept-mediated inhibition of TNF-α with reducing but still not completely reversed Ki67 counts after 1 week of treatment^112^. This indicates that hyperproliferation cannot be reverted in a prompt manner but slowly reverts over time.

Potent effects of the biological therapies were observed regarding inflammation suppression. Both, ADM as well as BMM monotherapies significantly decreased secretions of IL-8. ADM also reduced the release of IL-6. Additive effects were observed after combined TNF-α and IL-17 inhibition with normalized levels of IL-6 and IL-8 comparable to physiological conditions. In addition, the chemokine gene *CXCL1* was predominantly downregulated upon BMM treatment while ADM decreased the mRNA expression of the cytokine gene *IL1B*. TNF-α is a central immunological active mediator recruiting immune cells and stimulating the secretion of pro-inflammatory mediators like IL-6 and IL-8 by keratinocytes^122,123^. Inhibition of TNF-α has been shown to reduce the release of inflammatory mediators including IL-6, IL-8 and IL-1β in patients’ material^124–126^. Similar to the presented results, adalimumab was confirmed to potently reduce the mRNA expression of the *IL1B* gene in lesional tissues of PSO patients^127^. *CXCL1* appeared only marginally downregulated upon ADM treatment, which is in accordance to unaltered *CXCL1* mRNA levels found in biopsy samples of lesional PSO skin after 4 days of ADM treatment^128^. Another patient study, however, demonstrated a downregulation of *CXCL1* upon ADM treatment^129^. This aligns with the slight *CXCL1* downregulation measured in the present study. TNF-α has been shown to induce CXCL1 (GRO-α) while anti-TNF-α therapies led to an effective reduction of the CXCL1 (GRO-α) protein production^130^. A main regulator of chemokine genes like *CXCL1* and *CXCL8* is IL-17, which induces these chemoattractant molecules to recruit immune cells^119^. In accordance, reduced transcriptional levels of chemokine genes as well as reduced IL-8 secretions have been reported for anti-IL-17A therapies via BMM treatment^131^. Our data confirm reduced IL-8 secretions and a downregulated *CXCL1* gene expression upon BMM treatment of the PSO-HSE. Interestingly, a blockage of TNF-α for PSO treatment was associated with a decrease in Th17 subsets and IL-17 levels^132–134^. To include this effect in our treatment setting, a combination therapy of TNF-α inhibition via ADM and IL-17A/F blockage via BMM treatment was conducted. Of course, physicians very rarely prescribe dual biologic therapies due to the increased risk of side effects and sparse data availability. Cases of dual antibody treatment have been described for hard-to-treat PSO phenotypes with patients benefiting from the combination therapy^135^. The combination of ADM + BMM exerted a stronger anti-inflammatory activity. Especially, the secretion levels of IL-6 and IL-8 were potently decreased and the *CXCL1* gene expression was further repressed compared to monotherapies. This demonstrates the strong anti-psoriatic impact of a combined blockage of two disease-relevant mediators. In summary, anti-inflammatory effects of the biological therapies, which have been described in patient studies were re-producible in our in vitro PSO-HSE. This emphasizes their suitability as preclinical test tools.

With regard to AMP gene expression, ADM treatment of the PSO-HSE resulted only in slight reduction of *DEFB4A*, *S100A7*, *PI3* mRNA levels. However, the *LCN2* gene expression was significantly repressed upon the TNF-α antagonist. The gene expression of *DEFB4A* can be induced in keratinocytes via IL-17 and TNF-α synergistically^136–138^. Consistent with our result, a patient study confirmed a weak response of the *DEFB4A* expression to an anti-TNF-α therapy while the gene was strongly downregulated upon anti-IL-17A treatment^139^. Dual antibody therapy with ADM + BMM repressed the *DEFB4A* gene expression in the same manner as the BMM monotherapy did. This is indicative of IL-17A being the main regulator of *DEFB4A* gene expression rather than TNF-α. However, another study has shown positive effects via reduced β-defensin 2 protein levels after 6 weeks of treatment with a TNF-α antagonist^140^. Still, several studies provided evidence of sole IL-17-induced AMP expression of genes like *DEFB4A*, *PI3* and *LCN2*^111,141^. Hence, it is no surprise that anti-IL-17A therapies have been reported to reduce the elevated AMP gene expression in PSO samples^136,142,143^. In accordance to these in vivo data, BMM treatment efficiently downregulated the AMP gene expression of *DEFB4A*, *S100A7*, *PI3* and *LCN2* of the in vitro PSO-HSE supporting their close resemblance to in vivo skin reactions. Similar to the *DEFB4A* gene expression, *S100A7* and *PI3* were more potently inhibited by BMM but remained rather unresponsive to ADM treatment. No additive effects of AMP repression were observed when the PSO-HSE were treated with both ADM and BMM antibodies. This suggests IL-17A as primary inducer of keratinocyte AMP expression. BMM nearly completely reverted the high *S100A7* levels back to normal expression levels. On protein levels, none of the biological therapies did reduce the elevated S100A7 levels of the PSO-HSE after 6 days of treatment. Anti-TNF-α therapies (etanercept or adalimumab) can fail to normalize the elevated S100A7 in patients with PASI scores >15^144^. High levels of S100A7 correlate with disease severity and the resulting PASI score. This assumption is supported by Maurelli et al., who found higher psoriasin serum levels in patients with severe PSO than in those with mild manifestations of the disease^145^. The present result for in vitro ADM treatment is in accordance to these observations. The PSO-HSE seem to feature a severe form of PSO comparable to in vivo skin with PASI scores >15. Another patient study showed reduced S1007 levels via IHC staining after 6 weeks of etanercept treatment of PSO patients^140^. In the present study, a short-term treatment period of 6 days was executed, which might explain the absence of any alleviating effect on the S100A7 production. Inhibition of IL-17A/F has been demonstrated to restore physiological mRNA levels of *S100A7* in cellular and patient samples^146^. Consistently, BMM normalized the *S100A7* gene expression after treatment of the PSO-HSE. However, the regenerating effect was still not transferred to protein levels within the observation period. Anti-TNF-α and anti-IL-17A therapies have been reported to lower the PI3 production in psoriatic skin^147^. In accordance, the in vitro treatment with BMM reduced the *PI3* expression. ADM, in contrast, only slightly downregulated the mRNA levels of this AMP gene. The observed discrepancy to in vivo results might be explained by the afore mentioned effect of TNF-α reducing the Th17 signalling under in vivo conditions^132–134^. At least, dual blockage of TNF-α and IL-17A resulted in lower *PI3* mRNA expression in the present study. Furthermore, *LCN2* was downregulated upon ADM and BMM monotherapies as well as combination treatment. Biological therapies blocking TNF-α or IL-17A have been shown to reduce *LCN2* gene expressions after 12 weeks of patient treatment^114,148^. In regard of AMP gene expression, the PSO-HSE responded to biological treatment in a similar manner as in vivo skin, thus, successfully simulating treatment outcomes.

Cellular adhesion molecules contribute to the tissue integrity of the skin and its barrier function^149^. The synthesis of tight junctional cell-cell contact proteins like CLDN1/4, and TJP1 (ZO-1) can be inhibited by excessive IL-17 signalling leading to an impaired barrier formation^150^. Disrupted tight junctions result in a defective structure of the *stratum corneum*^151^. The PSO-HSE have previously been confirmed to feature an impaired horny layer^32^. Treatment with ADM did not improve the low mRNA expression of cell-cell contact genes. In contrast, IL-17A/F inhibition via BMM upregulated the gene expression of *DSG1* and *TJP1* and restored the *CLDN1* transcript levels. As shown by Lin et al., a restoration of cellular adhesion molecules can be associated with healing of psoriatic plaques^152^. Hence, an induction of cell-cell contact formation is not only essential to stabilize the barrier function but also supports the recovery of diseased tissue reverting to a physiological appearance. To the knowledge of the authors, previous studies with biological antibodies for PSO treatment have not focussed on their effects on cell-cell contacts. As suggested by our data and the study by Lin et al., disease-alleviating effects can also include improvements of cell–cell adhesions. These molecules should be taken into consideration as surrogate parameters to further evaluate the effectiveness of PSO therapies.

With the present study, in vitro skin models mimicking PSO have been proven to be a suitable 3D in vitro test environment for preclinical analyses. Topical treatments have been verified to exerted anti-inflammatory effects and AMP modulating properties. The present work is limited to a pilot study for the tested topical compounds. Other clinically relevant drugs for PSO therapy like retinoids, calcipotriol or JAK inhibitors were not screened. Specific blockage of PSO-mediating cytokines via biological therapies has been shown to have promising PSO-alleviating impacts. Here, anti-psoriatic effects were assessable upon the in vitro treatments despite immunological limitations. The present model can serve as a test tool for drugs targeting or interfering downstream the Th1/Th17 signalling and keratinocyte responses. Compounds blocking dendritic cell mediators, e.g. IL-23 inhibitors, cannot be reflected properly due to the procedure of PSO induction. As a proof of principle, the IL-23 antagonist risankizumab was tested with our setting and can be viewed in the Supplementary material (Supplementary Fig. 3-5), Therapeutic interventions are limited to cells present in the skin models, namely keratinocytes and fibroblasts. However, as long as therapeutic effects can be observed in these in vitro test systems, they will likely lead to positive effects under in vivo conditions.

## Material and methods

### 3D human skin equivalents and disease induction

The skin equivalents with a PSO phenotype were generated as described previously ^32^. In brief, the cutaneous tissues were assembled using primary dermal fibroblasts (purchased from PELO Biotech) and epidermal keratinocytes, which were obtained via in-house isolation from juvenile foreskin samples. The isolation procedure was performed in accordance with relevant guidelines and regulations provided and approved by the Ethics Committee of the Medical Faculty of the Friedrich Schiller University Jena (4739–03/16). All ethical regulations relevant to human research participants were followed. The legal guardians of all donors of primary keratinocytes submitted informed consent. The experiments were run with cells derived from one or two donors in total. All cells and skin equivalents were cultured at 37 °C under 5% CO_2_ and around 95% humidity. Dermal fibroblast were cultivated in Dulbecco’s modified Eagle’s medium (DMEM; AMIMED^®^ BioConcept Ltd.) containing 2% fetal bovine serum (FBS; PAN Biotech), 5 μg/mL recombinant human insulin (AMIMED^®^ BioConcept Ltd.), 5 ng/mL recombinant human fibroblast growth factor (Cellsystems) and 50 µg/mL gentamicin (Thermo Fisher). Adherent dermal fibroblasts were detached via trypsin-EDTA (Thermo Fisher) exposure. To generate the dermal compartment, 1.5 × 10^5^ cells were seeded per insert (0.4 µm pore diameter, Greiner Bio-One) placed in a 12-well plate. The dermal compartments were cultivated for 21 days in submersion medium consisting of DMEM with 10% FCS, 50 µg/mL gentamicin and 150 µg/mL ascorbic acid (Sigma Aldrich) with regular medium exchange. Before adding epidermal keratinocytes, the medium was removed and 75 µL of a 50 µg/mL fibronectin solution per insert were applied on top of the dermal compartments. Freezings of early primary keratinocyte passages were thawed and resuspended in keratinocyte growth medium 2 kit (KBM; PromoCell) supplemented with 10% FBS, 50 µg/mL gentamicin and 150 µg/mL ascorbic acid (Sigma Aldrich). A volume of 150 µL of a 9 ∙ 10^5^ keratinocytes/mL suspension was added per model. Five days later the calcium content of the medium was increased by additional supplementation with 1.88 mM CaCl_2_ (Serumwerk Bernburg AG). After 7 days of epidermis cultivation, the skin equivalents were exposed to an airlift cultivation by placing the inserts in 12-well ThinCert^®^ plates (Greiner Bio-One) and removing the medium on top of the skin models. During the airlift (AL) phase the cultivation medium changed to 1:1 DMEM + DMEM/F-12 (Thermo Fisher) customized with 5% FCS, 50 µg/mL gentamicin, 0.33 µg/mL hydrocortisone (Sigma Aldrich), 5 µg/mL transferrin (BBI Solutions), 5 µg/mL insulin (PELO Biotech), 3.99 × 10^6 ^ng/mL tri-iodothyronine (Sigma Aldrich), 13.51 µg/mL adenine (Sigma Aldrich), 1.88 mM CaCl_2_ and 150 µg/mL ascorbic acid. To induce the psoriatic phenotype, a recombinant cytokine cocktail was added to the culture medium every 2-3 days during the AL incubation. The PSO stimulation mix contained 10 ng/mL IL-17A, 10 ng/ml IL-6, 25 ng/mL IL-22, 10 ng/mL IL-1α and 10 ng/mL TNF-α (7Bioscience GmbH). The tissue generation of the skin equivalents finished at day 12 of AL incubation.

### Regeneration of 3D human skin equivalents with PSO

The regenerative potential of the PSO skin models was evaluated by removing the cytokine stimulus at day 6 of airlift incubation. By doing so, the following medium exchanges were performed using medium without cytokine stimulation to allow the skin models to recover for 6 days. Samples were taken at day 12 of airlift incubation.

### Treatment of 3D human skin equivalents with PSO

Stock solutions of dexamethasone (Sigma Aldrich) and celastrol were prepared in DMSO and further diluted in PBS to obtain a working concentration of 10 µM. For topical treatment, 75 µL of the test substances were applied on top of the tissue surface. Application of PBS only served as non-treatment control, while adding 0.2% DMSO was used for vehicle control. The treatment was performed during airlift incubation at day 0, 6 and 12 and samples were taken 24 h after the final treatment.

For subcutaneous therapy, the biolgicals adalimumab (Hyrimoz®, Hexal) and bimekizumab (Bimzelx®, UCB Pharma) were provided by Dr. med. Jörg Tittelbach. The antibodies were further diluted in AMPUWA® water for injection purposes (Fresenius Kabi) and respective volumes were added to the medium reservoir below the inserts resulting in concentrations of 10 µg/mL (adalimumab) or 80 µg/mL (bimekizumab). The antibodies were applied during airlift incubation of the skin models at day 6, 8 and 10 concomitant to the medium exchange and cytokine priming for PSO induction. Samples were taken at day 12 of airlift incubation.

### Histological sample preparation

The skin equivalent samples were transferred into embedding cassettes (Kabe Labortechnik) and incubated in 4% formaldehyde (Dr. K. Holborn & Söhne) for at least 24 h. The fixed skin models were paraffined using a tissue processing instrument (Tissue Processor TP1020, Leica). The tissues were embedded in paraffin blocks and sections of 4 µm were placed onto glass slides, which were then dried at 65 °C.

### Haematoxylin and eosin staining

The slides were deparaffined using xylene and rehydrated via gradually reduced ethanol concentrations (100% to 96% to 70% to aqua dest.). The slides were stained with hematoxylin (Gill II, Merck) and 0,5% eosin (Merck) and dehydrated using increasing ethanol concentrations to xylene. Afterwards, the slides were mounted with Histofluid® mounting medium (Marienfeld).

### Immunohistological staining

The slides were deparaffined as described before. For specific protein detection, the following antibodies were chosen: anti-human Ki67 (Biozol, Abnova), anti-human filaggrin, anti-human S100A7, anti-human cytokeratin 16 (Thermo Fisher Scientific) and anti-human cytokeratin 10 (Santa Cruz Biotechnology). Heat-induced epitope retrieval was performed by incubating the slides in pre-heated Tris-EDTA with 0.05% Tween in a steamer for 30 min. The chilled slides were rinsed in TBS with 0.025% Triton X-100 for 2 × 5 min. All samples were incubated in UltraCruz^®^ Blocking Reagent (Santa Cruz Biotechnology) for 30 min in a humidity chamber. Dilutions of the primary antibodies (FLG 1:200; Ki67 1:40; S100A7 1:4000; CK16 1:150; CK10 1:1000 in Blocking Reagent) were added to the slides and incubated overnight at 4 °C in a humidity chamber. The slides were rinsed again in TBS with 0.025% Triton X-100 for 2 × 5 min. The biotinylated secondary antibody (goat anti-mouse IgG, Biozol) was diluted (1:500 for FLG, Ki67, CK16, CK10 or 1:1000 for S100A7) and added to the slides. After 1 h of incubation, the slides were rinsed again in TBS with 0.025% Triton X-100 and the prepared avidin-biotin complex conjugated with alkaline phosphatase (VECTASTAIN^®^ ABC-AP Kit) was applied and incubated for 30 min in a humidity chamber. After another washing step, the substrate solution (Fast Red Substrate Kit, Abcam) was prepared according to the manufacturer’s instructions and added to the slides. The incubation period varied depending on the IHC target (CK16, FLG 15 min; Ki67 10 min; CK10, S100A7 5 min) followed by another TBS-Tween washing procedure and a rinsing step with dH_2_O. The samples were stained with hematoxylin Gill I for 30 s and blueing of nuclear chromatin was obtained by rinsing the slides with tap water. After washing in dH_2_O, the slides were covered using Aquatex^®^ mounting medium.

### ELISA (IL-6, IL-8/CXCL8)

Protein secretion analysis of IL-6 and IL-8/CXCL8 in the undernatants of the skin equivalents were carried out using the IL-6 ELISA development kit (Mabtech) and IL-8/CXCL8 DuoSet ELISA (R&D Systems) according to the manufacturer’s instructions. Briefly, 100 µL of the standard series and diluted supernatants were added to high binding plates covered with capture antibodies and incubated for 2 h. After washing, a biotinylated detection antibody was added and incubated for 1 h (IL-6 ELISA) or 2 h (IL-8 ELISA) followed by another washing procedure. Afterwards, streptavidin-HRP was applied for 1 h (IL-6 ELISA) or 20 min (IL-8 ELISA) and plates were washed again before adding TMB solution (1:1 TMB substrate + hydrogen peroxide; Thermo Fisher Scientific). The reaction was terminated by the addition of 2 M sulfuric acid and absorbance was measured at 450 nm and reference reading at 620 nm. The concentration of the secreted target proteins was calculated by a four-parameter fitting with logarithmic scaled concentrations and linear scaled OD values. The endogenous IL-6 secretion levels were corrected for the PSO samples by subtraction of IL-6 levels measured for the sole PSO medium sample containing the recombinant IL-6. This medium blank sample was incubated concomitantly to the skin equivalents during the experimental process.

### RNA extraction, cDNA synthesis, real-time quantitative PCR

Gene expression analysis was performed as mentioned previously ^32^. RNA was isolated in accordance with the guidelines provided by the RNeasy Mini kit (Qiagen). The tissue samples underwent lysis in RLT buffer with 10 µL/mL 2-mercaptoethanol and homogenization with 3 mm steal beads at 30 Hz. Proteinase K (AppliChem) was applied to the samples and incubated at 55 °C with shaking at 1000 rpm for 10 min. The extraction of the endogenous RNA was performed using the QIAcube robotic workstation. Genomic DNA residues were removed upon DNase I treatment using the DNase I, RNase-free kit (Thermo Fisher) following the manufacturer’s instructions. RNA concentrations were measured and diluted to 40 ng/µL, which were used as templates for cDNA synthesis. Reverse transcription was carried out using the High capacity cDNA Reverse Transcription kit (Thermo Fisher) in accordance with the provided instructions. Real-time PCR (qPCR) was performed using the QuantiNova™ SYBR Green PCR Kit (Qiagen). Samples with a cDNA concentration of 0.5 ng/µL were adjusted and used for the PCR reaction. The reaction mix was prepared in 20 µL reaction volumes consisting of 1x SYBR Green master mix, 500 nM forward primer, 500 nM reverse primer and 3 µL of the diluted cDNA. The real-time target amplification was performed in a qTOWER^3^G (Analytik Jena) cycler running the following programme: 95 °C for 3 min (heat activation of the polymerase), 40 cycles of 95 °C for 5 s (denaturation), 57 °C for 10 s (primer annealing), 72 °C for 10 s (extension). Primer efficiencies were determined by standard curve analyses and used for an expression ratio quantification via the algorithm by Pfaffl et al.^153^. Target gene expression was normalized to reference expression of the housekeeping gene *ACTB*. Expression values were log_2_ transformed with log_2_ (2) = 1 referring to a twofold upregulation and log_2_ (0.5) = −1 correlating with a twofold downregulation. Primer sequences are listed in Table 1.

**Table 1:** Primer sequences or purchase information of used oligonucleotides

Target gene	Primer sequence or order ID	Manufacturer
*ACTB*	fw:5‘-TGCCGACAGGATGCAGAAG-3’ rev:5‘-CTCAGGAGGAGCAATGATCTTGA-3‘	Eurofins
*CLDN1*	Hs_CLDN1_1_SG QuantiTect® Primer Assay	QIAGEN
*COX2*	fw:5‘-GCAATAACGTGAAGGGCTGTC-3‘ rev:5‘-AGCGGGAAGAACTTGCATTG-3‘	Eurofins
*CXCL1*	fw: 5‘-TCACCCCAAGAACATCCAAAG-3‘ rev: 5‘-GAGTGTGGCTATGACTTCGGTTT-3‘	Eurofins
*CXCL8*	fw: 5‘-TTCTAGGACAAGAGCCAGGAAG-3’ rev: 5‘-AAATCAGGAAGGCTGCCAAG-3‘	Eurofins
*DEFB4A*	fw: 5‘-TGATGTCCTCCCCAGACTCA-3’ rev: 5‘-CCACCAAAAACACCTGGAAGAG-3‘	Eurofins
*DSG1*	fw:5‘-TCCCCACATTTCGGCACTAC-3‘ rev: 5‘-GCCCAGAGGATCGAGAATAGG-3‘	Eurofins
*FLG*	fw:5‘-AGGACACAAGCACAGAAAGC-3’ rev:5‘-TCTCTTGGGCTCTTGGATCTTC-3‘	Eurofins
*IL1B*	fw: 5‘-GGACAAGCTGAGGAAGATGC-3‘ rev: 5‘-TCCATATCCTGTCCCTGGAG-3‘	Eurofins
*IL23A*	fw:5‘-GAATCAGGCTCAAAGCAAGTGG-3’ rev:5‘-AGCAACAGCAGCATTACAGC-3‘	Eurofins
*IL6*	fw: 5‘-CCACCGGGAACGAAAGAGAA-3‘ rev: 5‘-GAGAAGGCAACTGGACCGAA-3‘	Eurofins
*KRT10*	fw: 5‘-GGGACCAAGATACTAACAAAACC-3‘ rev: 5‘-TGAAAGAACTCTACCGTCGGG-3‘	Eurofins
*LCN2*	fw: 5‘-ATGTCACCTCCGTCCTGTTTAG-3‘ rev: 5‘-TAATGTTGCCCAGCGTGAAC-3‘	Eurofins
*PI3*	Hs_PI3_2_SG QuantiTect® Primer Assay	QIAGEN
*S100A7*	fw: 5′-GTCCAAACACACACATCTCACT-3′ rev: 5′-TCATCATCGTCAGCAGGCTT-3′	Eurofins
*TJP1*	Hs_TJP1_1_SG QuantiTect® Primer Assay	QIAGEN

Real-time PCR analyses were carried out using the following oligonucleotides for target gene amplification and quantification. Forward (fw) and reverse (rev) primer are stated in 5’–3’ direction or order information was given in case of used Qiagen products.

### Statistics and reproducibility

The IBM SPSS Statistics 26 software was used for statistical evaluation. Error bars of the bar charts represent standard deviations (SD). Data were analysed regarding normal distribution by Kolmogorov–Smirnov test. Data with normal distribution were further processed using the one-way ANOVA. The post hoc test was chosen according to the homogeneity of variance. The Bonferroni post hoc test was applied for data with homogeneous variances while Dunett’s T3 post hoc was used for data sets with inhomogeneous variances. Nonparametric data sets were statistically analysed using the Mann–Whitney *U* test. Significant deviations were defined by *p* ≤ 0.05 *, *p* ≤ 0.01 **, *p* ≤ 0.001 ***. The data were derived from sample sizes of *n* = 2 up to *n* = 6 skin equivalents. The respective sample size for each experimental setting is stated in the figure legends. Each sample was measured in technical duplicates for ELISA, gene expression analysis or LDH release.

### Reporting summary

Further information on research design is available in the Nature Portfolio Reporting Summary linked to this article.

## Supplementary information

Supplementary Information

Description of Additional Supplementary Materials

Supplementary Data 1

Reporting Summary

## Data Availability

All numerical data generated or analysed during this study are included in the Supplementary Data 1 file.
