# Supplementary material for: Bilayered skin equivalent mimicking psoriasis as predictive tool for preclinical treatment studies
Source: Commun Biol. 2024 Nov 18;7:1529. doi: 10.1038/s42003-024-07226-x (PMC11574237; doi:10.1038/s42003-024-07226-x)
Supplement: Supplementary file 1 — Supplementary Information [file 42003_2024_7226_MOESM1_ESM.pdf]

SUPPLEMENTARY

Additional data for topical treatment experiments

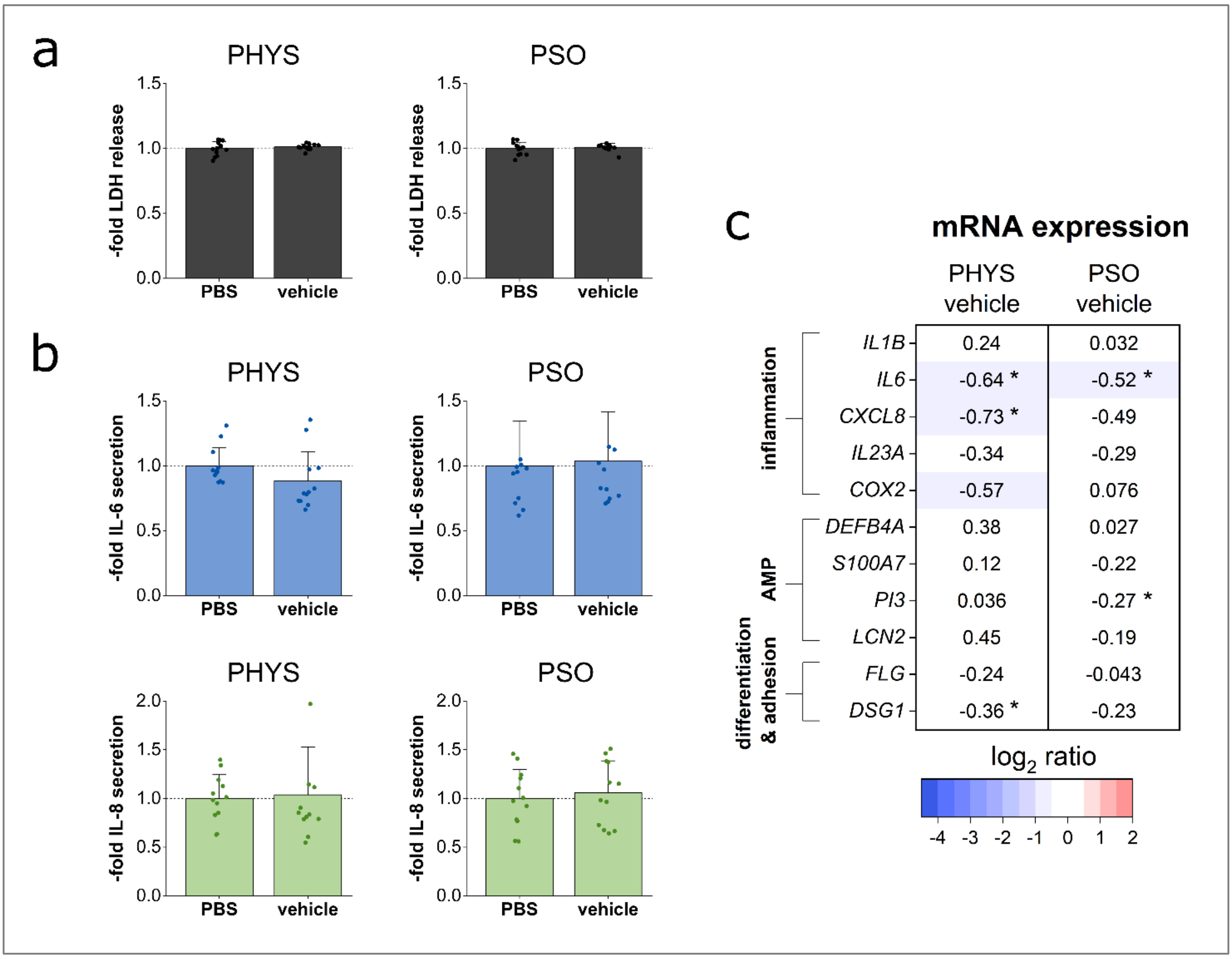

Supplementary Figure 1: Exclusion of treatment effects by the DMSO vehicle.
(a) Cytotoxic effects were excluded by analysis of LDH secretion compared to untreated samples. Statistics: one-way ANOVA with Dunett's T3 post hoc test; p≤0.05 *, p≤0.01 **, p≤0.001 *** compared to untreated PBS control, n=6 skin equivalents measured in technical duplicates. (b) Protein secretions of IL-6 (blue) and IL-8 (green) in the undernatants of the organoids at day 12 were measured by ELISA. Statistics: Mann-Whitney U test; p ≤ 0.05 *, p ≤ 0.01 **, p ≤ 0.001 *** compared to untreated PBS control, n=6 skin equivalents measured in technical duplicates. (c) Gene expression was analysed via qPCR. Genes of interest include genes encoding inflammatory markers like interleukin 1β (*IL1B*), interleukin 6 (*IL6*), chemokine (CXC motif) ligand 8 (*CXCL8*), interleukin 23A (*IL23A*) and cyclooxygenase-2 (*COX2*), genes encoding antimicrobial peptides (AMP) like beta-defensin-2 (*DEFB4A*), psoriasin (*S100A7*), elafin (*PI3*) and lipocalin-2 (*LCN2*) and genes encoding the differentiation marker filaggrin (*FLG*) or the desmosomal cell-cell-contact protein desmoglein-1 (DSG1). Expression values relative to the untreated PBS control of PHYS or PSO models were $\log_2$ transformed. This transformation leads to a mean $\log_2$ value = 0 for PBS control (not shown). Upregulations of mRNA levels upon treatment are shown in red for values > 0 while downregulations are depicted in blue for values < 0. Statistics: Mann-Whitney U test; p ≤ 0.05 *, p ≤ 0.01 **, p ≤ 0.001 *** compared to the corresponding untreated PBS control, n=5 skin equivalents measured in technical duplicates.

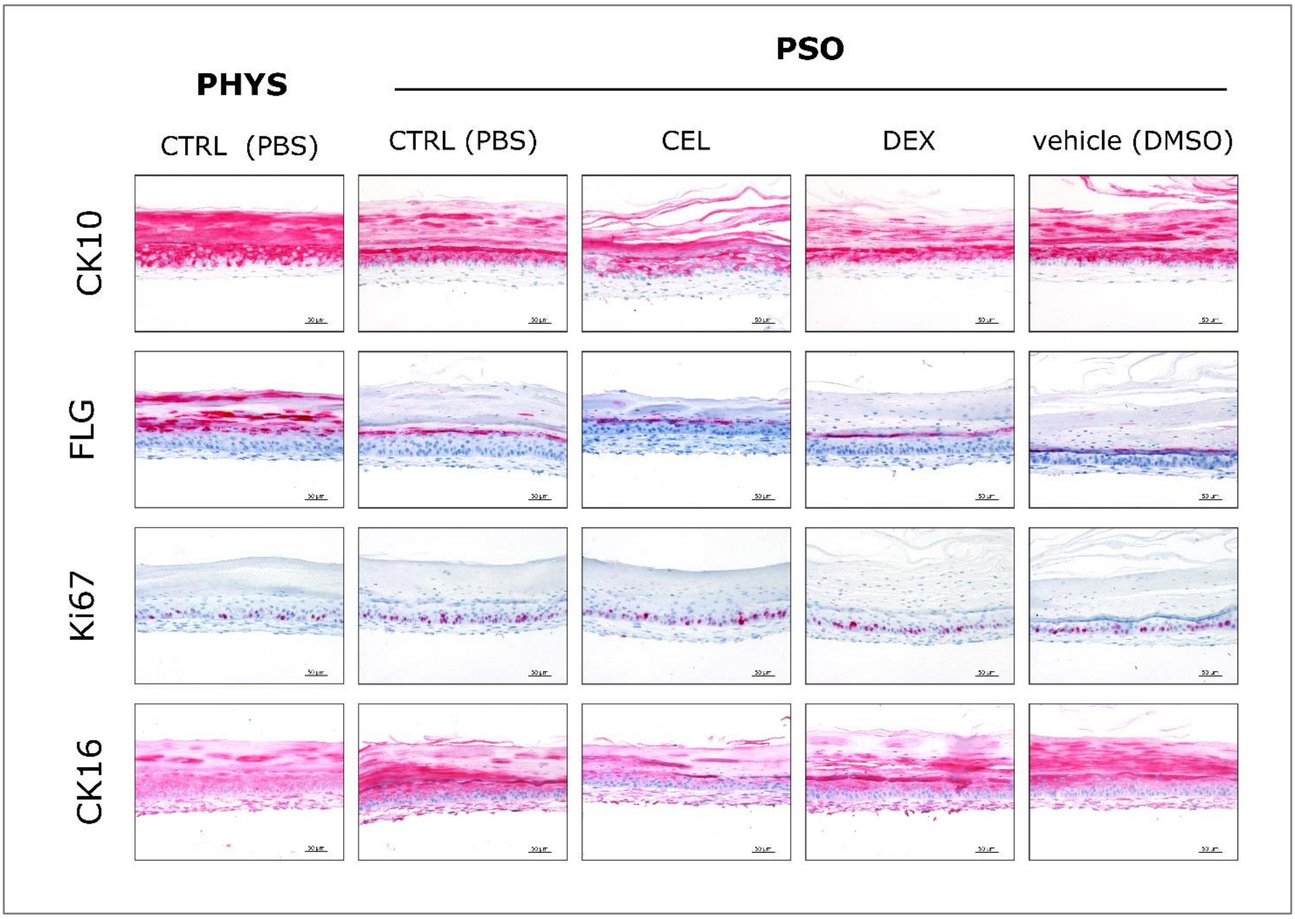

Supplementary Figure 2: Topical treatment effects on protein expression of PSO-HSE.
3D skin equivalents were cytokine-primed for psoriasis (PSO) induction and celastrol (CEL) or dexamethasone (DEX) were applied topically at day 0, 6 and 12 of airlift incubation. Effects on protein production of the differentiation markers keratin-10 (CK10), filaggrin (FLG), the mitosis marker Ki67 and pathological keratin-16 (CK16) were analysed by IHC. The protein of interest is stained in red by IHC processing. Scale bar: 50 µm.

Additional data for a biological control compound targeting a mediator of dendritic cells

Risankizumab (used product: Skyrizi®, AbbVie) is a monoclonal antibody inhibiting IL-23A, a cytokine released by dendritic cells to induce the polarization of Th17 cells. The PSO models in the present study were generated by simulating the pathological Th1/Th17 signalling following the dendritic signalling. Hence, risankizumab was tested as control biologic. Here, we hypothesized no PSO-alleviating effects since the IL-23 signalling itself is not included in the cytokine stimulation mix.

Risankizumab (RSM) did not lead to morphological changes or an increased LDH release. Hence, no cytotoxic effects were observed (Supplementary Fig. 3). As hypothesized, the pathological protein alterations of CK10, CK16, FLG, S100A7 and Ki67 remained unaffected under RSM therapy compared to the untreated PSO control (Supplementary Fig. 4b). The secretion of IL-8/CXCL8 was also not reduced by RSM. Interestingly, a potent decline in the IL-6 secretion was measured after RSM treatment of the PSO models (Supplementary Fig. 4a). As shown in Figure 3, the keratinocytes of the PSO models expressed increased mRNA levels of the *IL23A* gene. Thus, we concluded that risankizumab blocked the keratinocyte IL-23 resulting in lower levels of IL-6. A study by Lindroos et al. confirmed that IL-23 is an inducer of the IL-6 secretion correlating with psoriatic hyperplasia (Lindroos et al., Invest Dermatol 2011)[1].
As expected, treatment of the PSO skin models with RSM did not revert the pathological changes of the PSO stimulation since none of the mediators present in the stimulation mix was blocked by the IL-23 inhibitor. Consequently, the expression of the AMP genes *DEFB4A*, *S100A7*, *PI3* and *LCN2* and the inflammatory genes *CXCL1* and *IL1B* were not decreased. In addition, RSM neither induced the low psoriatic mRNA levels of the differentiation markers *FLG* or *KRT10* nor was the expression of cell-cell contact genes like *CLDN1*, *DSG1* and *TJP1* enhanced.

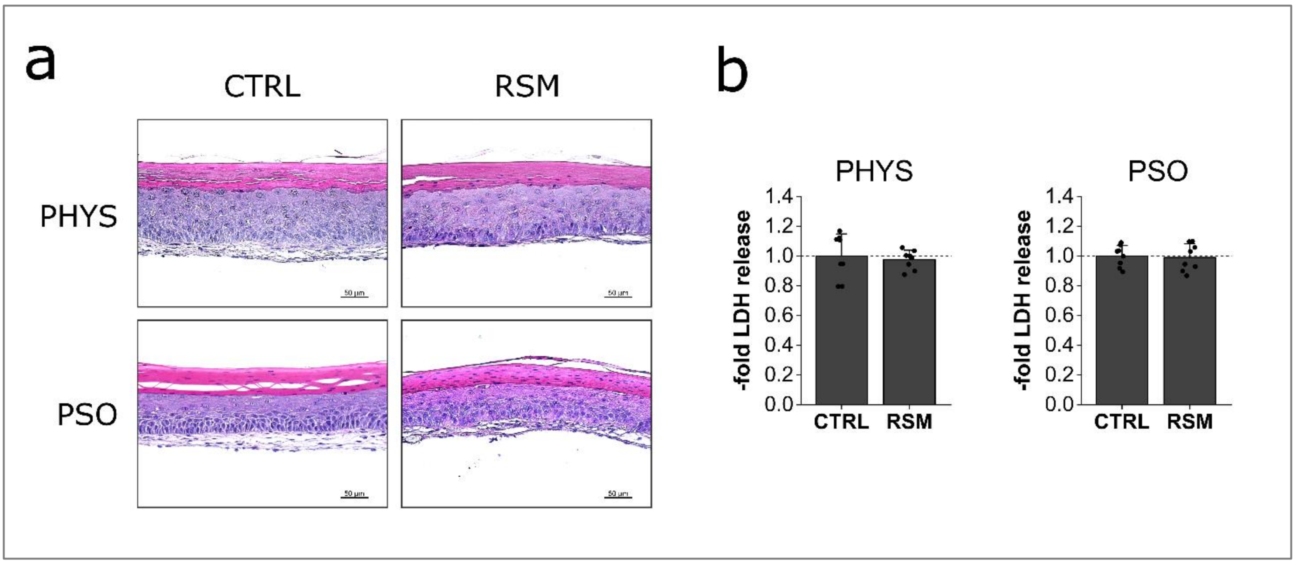

Supplementary Figure 3: *In vitro* skin compatibility of the IL-23 inhibitor risankizumab for treatment of PSO-HSE.
3D skin equivalents were cultivated physiologically (PHYS) or cytokine-primed for psoriasis (PSO) induction. Risankizumab (RSM), a humanized monoclonal antibody targeting IL-23 was applied subcutaneously at day 6, 8 and 10 of airlift incubation. The concentration of the antibody in the medium reservoir of the skin models was 37.5 µg/mL. (a) HE staining was used to reveal putative cytotoxic effects via morphological changes after treatment of PHYS or PSO-HSE. Scale bar: 50 µm, representative pictures of n=2 skin equivalents. (b) Cytotoxic effects were further excluded by analysis of LDH secretion compared to untreated samples. Statistics: one-way ANOVA with Dunett's T3 post hoc test; $p \leq 0.05$ *, $p \leq 0.01$ **, $p \leq 0.001$ *** compared to untreated control, n=4 skin equivalents measured in technical duplicates.

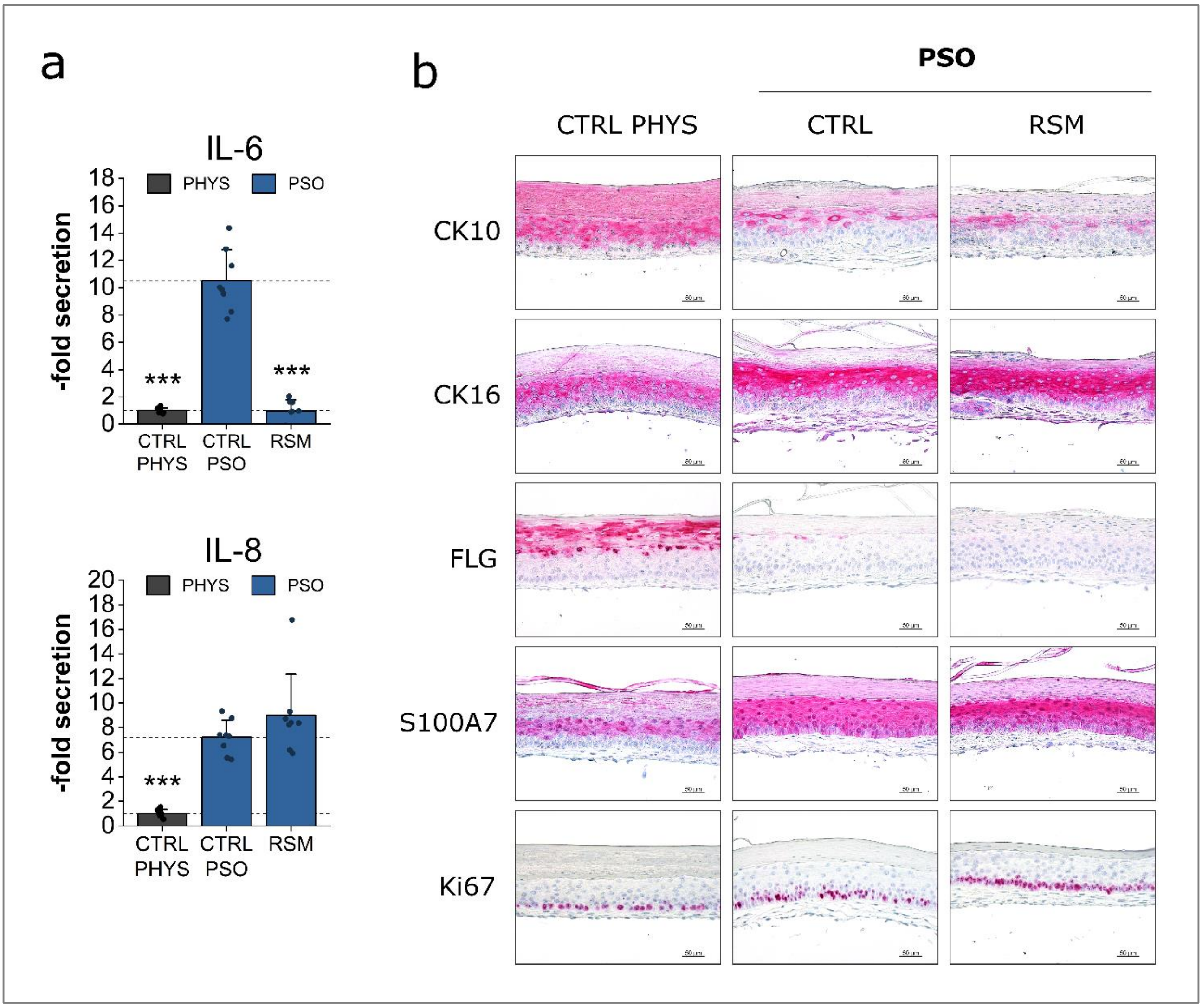

Supplementary Figure 4: Risankizumab treatment effects on cytokine secretion and protein expression.

3D skin equivalents were cultivated physiologically (PHYS) or cytokine-primed for psoriasis (PSO) induction. Risankizumab (RSM), a humanized monoclonal antibody targeting IL-23 was applied subcutaneously at day 6, 8 and 10 of airlift incubation. The concentration of the antibody in the medium reservoir of the skin models was 37.5 µg/mL. (a) Protein secretions of IL-6 and IL-8 were measured by ELISA to investigate anti-inflammatory properties of the antibody therapy. Statistics: Mann-Whitney U test; $p \leq 0.05$ *, $p \leq 0.01$ **, $p \leq 0.001$ *** compared to untreated PSO control, n=4 skin equivalents measured in technical duplicates. (b) Effects on protein production of the differentiation markers keratin-10 (CK10), filaggrin (FLG), pathological keratin-16 (CK16), the antimicrobial peptide psoriasin (S100A7) and the mitosis marker Ki67 were analysed by IHC. The protein of interest is stained in red by IHC processing. Scale bar: 50 µm, representative pictures of n=2 skin equivalents

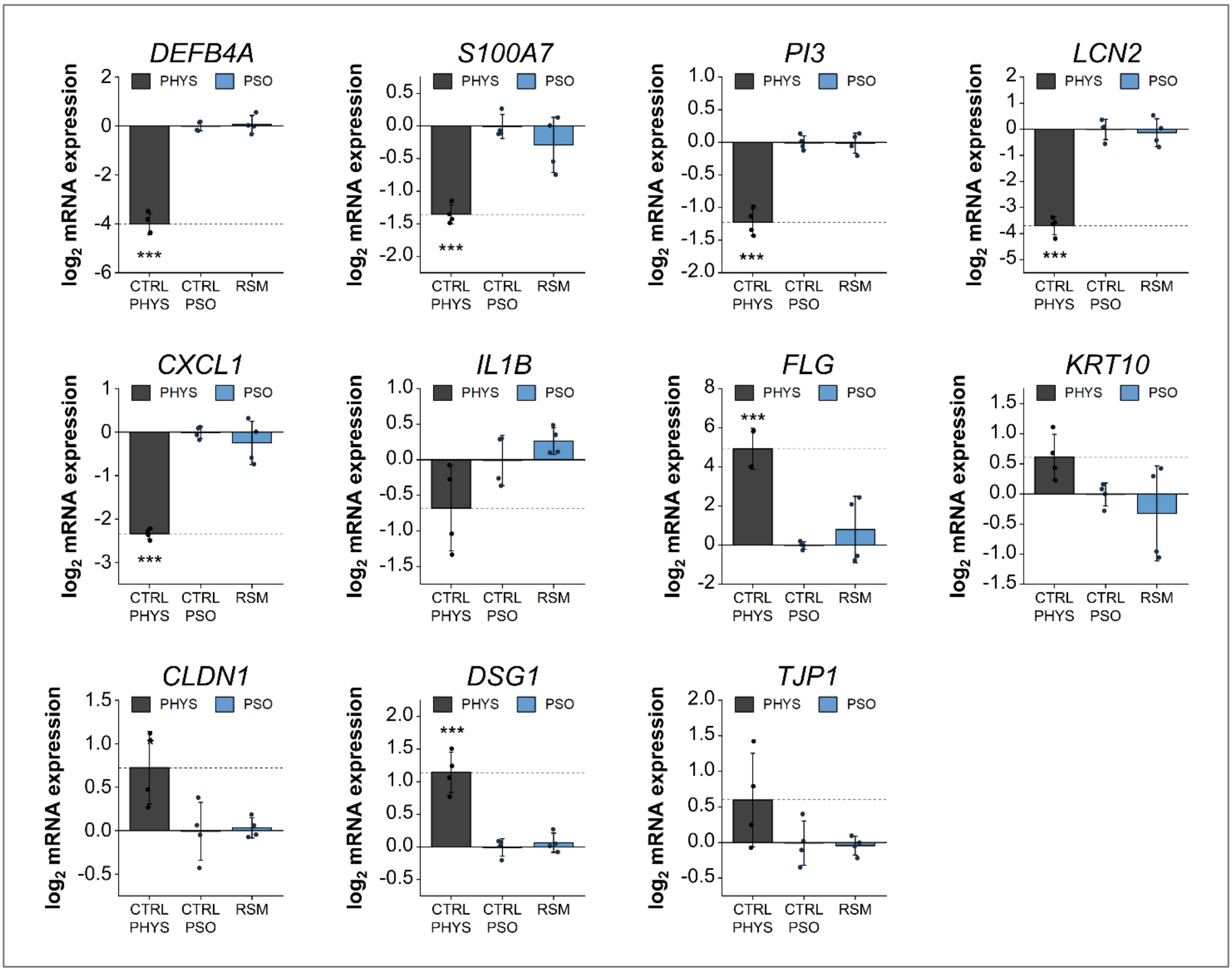

Supplementary Figure 5: Risankizumab treatment effects on gene expression.
3D skin equivalents were cultivated physiologically (PHYS) or cytokine-primed for psoriasis (PSO) induction. Risankizumab (RSM), a humanized monoclonal antibody targeting IL-23 was applied subcutaneously at day 6, 8 and 10 of airlift incubation. The concentration of the antibody in the medium reservoir of the skin models was 37.5 µg/mL. Gene expression was analysed via qPCR. The mRNA expressions of genes altered in PSO milieu (blue) were investigated to examine reverting effects under biological treatment in relation to physiological gene expression (grey). Genes of interest include genes encoding antimicrobial peptides (AMP) like beta-defensin-2 (*DEFB4A*), psoriasin (*S100A7*), elafin (*PI3*) and lipocalin-2 (*LCN2*), genes encoding inflammatory markers like chemokine (CXC motif) ligand (*CXCL1*) and interleukin 1β (*IL1B*), genes encoding the differentiation markers filaggrin (*FLG*) and keratin-10 (*KRT10*) or genes encoding cell-cell contact proteins like claudin-1 (*CLDN1*), desmoglein-1 (*DSG1*) and tight junction protein-1 (*TJP1*). Expression values relative to the untreated PSO control were $\log_2$ transformed. This transformation leads to a mean $\log_2$ value = 0 for the untreated PSO control. Upregulations of mRNA levels upon treatment are indicated by values > 0 while downregulations are indicated by values < 0. Statistics: one-way ANOVA with Bonferroni post hoc test; $p \leq 0.05$ *, $p \leq 0.01$ **, $p \leq 0.001$ *** compared to untreated PSO control, n=2 skin equivalents measured in technical duplicates.

Reference:

1 Lindroos, J. *et al.* IL-23-mediated epidermal hyperplasia is dependent on IL-6. *J Invest Dermatol* **131**, 1110-1118, doi:10.1038/jid.2010.432 (2011).
