# Supplementary material for: Bilayered skin equivalent mimicking psoriasis as predictive tool for preclinical treatment studies
Source: Commun Biol. 2024 Nov 18;7:1529. doi: 10.1038/s42003-024-07226-x (PMC11574237; doi:10.1038/s42003-024-07226-x)
Supplement: Supplementary file 2 — Description of Additional Supplementary Materials [file 42003_2024_7226_MOESM2_ESM.pdf]

Description of Additional Supplementary Files

File name: Supplementary Data 1

Description: Numerical source data
